# Long-term strict ant-plant mutualism identity characterises growth rate and leaf shearing resistance of an Amazonian myrmecophyte

**DOI:** 10.1038/s41598-024-67140-4

**Published:** 2024-08-01

**Authors:** Rafael E. Cárdenas, Camila Rodríguez-Ortega, Daniel Utreras, Dale L. Forrister, María-José Endara, Simon A. Queenborough, Pablo Alvia, Pablo A. Menéndez-Guerrero, Selene Báez, David A. Donoso

**Affiliations:** 1 Museo de Zoología QCAZ, Laboratorio de Entomología y Herbario QCA, Laboratorio de Ecología de Plantas, Escuela de Ciencias Biológicas, Facultad de Ciencias Exactas y Naturales, Pontificia Universidad Católica del Ecuador, Av. 12 de Octubre 1076 y Roca, Apdo. 17-01-2184, Quito, Ecuador; 2 Smithsonian Tropical Research Institute, Apdo. 0843-03092, Balboa, Republic of Panama; 3 Grupo de Investigación en Ecología Evolutiva en los Trópicos-EETROP, Universidad de Las Américas, Quito, Ecuador; 4 Yale School of the Environment, Yale University, New Haven, CT USA; 5 Estación Científica Yasuní, Pontificia Universidad Católica del Ecuador, Parque Nacional Yasuní, Orellana, Ecuador; 6 Laboratorio de Macroecología y Cambio Global, Facultad de Ciencias Exactas y Naturales, Pontificia Universidad Católica del Ecuador, Av. 12 de Octubre 1076 y Roca, Apdo. 17-01-2184, Quito, Ecuador; 7 Departamento de Biología, Facultad de Ciencias, Escuela Politécnica Nacional, Quito, Ecuador

**Keywords:** Behavioural ecology, Community ecology, Tropical ecology

## Abstract

Over 125 million years of ant-plant interactions have culminated in one of the most intriguing evolutionary outcomes in life history. The myrmecophyte *Duroia hirsuta* (Rubiaceae) is known for its mutualistic association with the ant *Myrmelachista schumanni* and several other species, mainly *Azteca*, in the north-western Amazon. While both ants provide indirect defences to plants, only *M. schumanni* nests in plant domatia and has the unique behaviour of clearing the surroundings of its host tree from heterospecific plants, potentially increasing resource availability to its host. Using a 12-year survey, we asked how the continuous presence of either only *M. schumanni* or only *Azteca* spp. benefits the growth and defence traits of host trees. We found that the continuous presence of *M. schumanni* improved relative growth rates and leaf shearing resistance of *Duroia* better than trees with *Azteca*. However, leaf herbivory, dry matter content, trichome density, and secondary metabolite production were the same in all trees. Survival depended directly on ant association (> 94% of trees died when ants were absent). This study extends our understanding of the long-term effects of strict ant-plant mutualism on host plant traits in the field and reinforces the use of *D. hirsuta*–*M. schumanni* as a model system suitable for eco-co-evolutionary research on plant–animal interactions.

## Introduction

Arboreal ant nesting originated during the Mesozoic era (late Cretaceous, around 78 mya) and developed first in lineages that began to forage on trees (mid-early Cretaceous, ~ 125 mya), and that later relied partially or fully on a plant-derived diet (~ 108 mya)^1,2^. Such an intrinsic relationship between plants and ants occurred before the evolution by plants of ant-associated structures, such as elaiosomes or domatia, and was a fundamental key to the diversification of early flowering plants^1^. As angiosperms began their ecological dominance of Earth, plants and ants evolved more interdependent associations with each other: resulting in symbiotic mutualisms that drive the structure and function of many ecosystems^3^. Mutualisms, reciprocally beneficial interactions between two species, are pervasive in nature and are fundamentally important at all levels of biological organisation because they allow organisms to excel in otherwise marginal habitats, reduce competition, exploit new niches, and buffer environmental variability^4,5^. Classic mutualism is found in the associations between ants and plants^6^ in which ants are generally attracted by specific plant structures (e.g., food bodies, extrafloral nectaries, or shelter) and provide important indirect defence against herbivory, pathogens, or intrusive vegetation^6–8^; but ant species presence in a plant may not always be an indicator of efficacy against herbivores, e.g.,^9^. These interactions range from opportunistic, to facultative associations, to specific obligate symbioses. While facultative ant-plant interactions are common in every terrestrial ecosystem except for the arctic regions, obligate interactions are limited to tropical regions^10–12^. Notably, obligate ant-plant interactions are often associated with domatia-bearing plants (known as myrmecophytes) that house ant colonies in hollow, swollen structures: branches, thorns, petioles, or leaf pouches^6,7,13^.

While there is abundant evidence showing that ant symbionts provide defensive advantages to their host plants, including hetero- and conspecific neighbours^14–16^, benefits are also often linked to roles that include the supply of resources (e.g., water, light or nutrients) central for development and survival. After seminal studies by Thompson^17^, the use of isotope tracers demonstrated that plants can engage in myrmecotrophy, exploiting resources derived from ant activity. For example, soil beneath plants with ant nests contains significantly higher concentrations of nitrate, ammonium, phosphorus, and water^18^. Such debris and waste concentrated by ants can be a supplemental source of nutrients for the plant^7,19–22^. As the nutrients are moved from ant mounds to leaf tissues, trees that host ant nests may grow more, have higher fruit set, and can produce nearly twice as many seeds as trees without nests^16^ and references therein; ^18,21,23–25^. In addition to nutrients, light availability has a direct effect on plant survival. For example, Yamawo et al.^26^ showed experimentally that better light conditions increased the development of extrafloral nectaries, increased the number of ants on the plants, and hence indirectly provided better protection against herbivores. However, plant defence against herbivory comes at a cost^27^, directly by reducing the resources available for growth or reproduction, or indirectly by reducing competitiveness: trade-offs where both are reduced by investment in defence. Thus, better nourished plants may invest more in defences (constitutive or induced), as well as in structures such as domatia or nectaries that are attractive to the protective ants. Testing variation by host plants in investment to defence, growth, or reproduction, and how these trade-offs are mediated by mutualist ants is often accomplished with a species-comparison approach where different mutualist pairs are examined. However, since different species are likely to provide different benefits to host plants, a plant that hosts many ant species is likely a better model for comparisons.

*Duroia hirsuta* (Poepp. & Endl.) K.Schum., 1888 (Rubiaceae), a plant species of the northwestern Amazon that engages in mutualistic associations with ants, is best known for the formation of monospecific stands known as devil’s gardens (*owekawente* in Waorani, and *supay-chakras* in Kichwa local languages) (RC pers. obs;^14,28^). Such monospecific stands are only formed when this plant species is in association with the ant species *Myrmelachista schumanni* Emery, 1890 (Formicidae) (also known as ‘lemon ant’;^29^). Apart from protecting its host plant against herbivory, this ant species injects formic acid into heterospecific plants in the immediate surroundings of their host tree, leading to large clearings in the forest^7,28,30–32^. Devil’s gardens in the Yasuní forest of Ecuador may typically comprise a maximum of 11 individuals (1–2 adult trees + seedlings and saplings)^14^, but larger monospecific stands have been reported in the Peruvian Amazon, with up to 594 *Duroia* trees, 3 million ant workers, and 15,000 queens^14,28,31^. This clearing behaviour reduces competition for the 1–2% of light reaching the understory for young *Duroia* growing in devil’s gardens^14,30^. However, outside devil’s gardens, *D. hirsuta* trees can be found in association with ants of other species, such as *Azteca* spp. These common and abundant predatory Amazonian ants nest and forage in mosaics in surrounding trees and do provide protection from herbivores to their host *D. hirsuta*, although they do not clear heterospecific plants from the local area^31^ (mosaics, defined as "the presence of large ant territories in the canopy, resulting from the patrolling of dominant and aggressive ant species which alternate, one with the other, among the tree crowns"^33^ and references therein). Both *D. hirsuta* and *Azteca* spp. have a high index of specialization (0.77 and 0.72 respectively, on a scale from 0 to 1;^34,35^). While *M. schumanni* nests in *D. hirsuta, Azteca* spp. does not, but maintain continuous high foraging activity on *D. hirsuta* trees^14,34^. Since *Azteca* spp. symbiosis with *D. hirsuta* has been documented as a true mutualism^31^ despite nesting in other neighbouring trees, we will keep this concept throughout the manuscript.

In this study, we determined whether *D. hirsuta* individuals in mutualistic monospecific long-term interactions with ants of either *M. schumanni* or *Azteca* spp. differed in their life-history traits. In particular, we examined relative growth rate, investment in physical and chemical defences, and survival of the host trees. We hypothesised that trees with the mutualistic *M. schumanni* may potentially benefit from additional resources (water, light, and nutrients, provided by the clearings) that trees with *Azteca* spp. do not have. These extra resources would allow the trees hosting *M. schumanni* to invest more in growth and defence, resulting in faster growth rates, more direct and indirect physico-chemical defences, and lower herbivory damage compared to trees hosting *Azteca* spp.

## Methods

### Study site and sample collection

**Figure 1:**
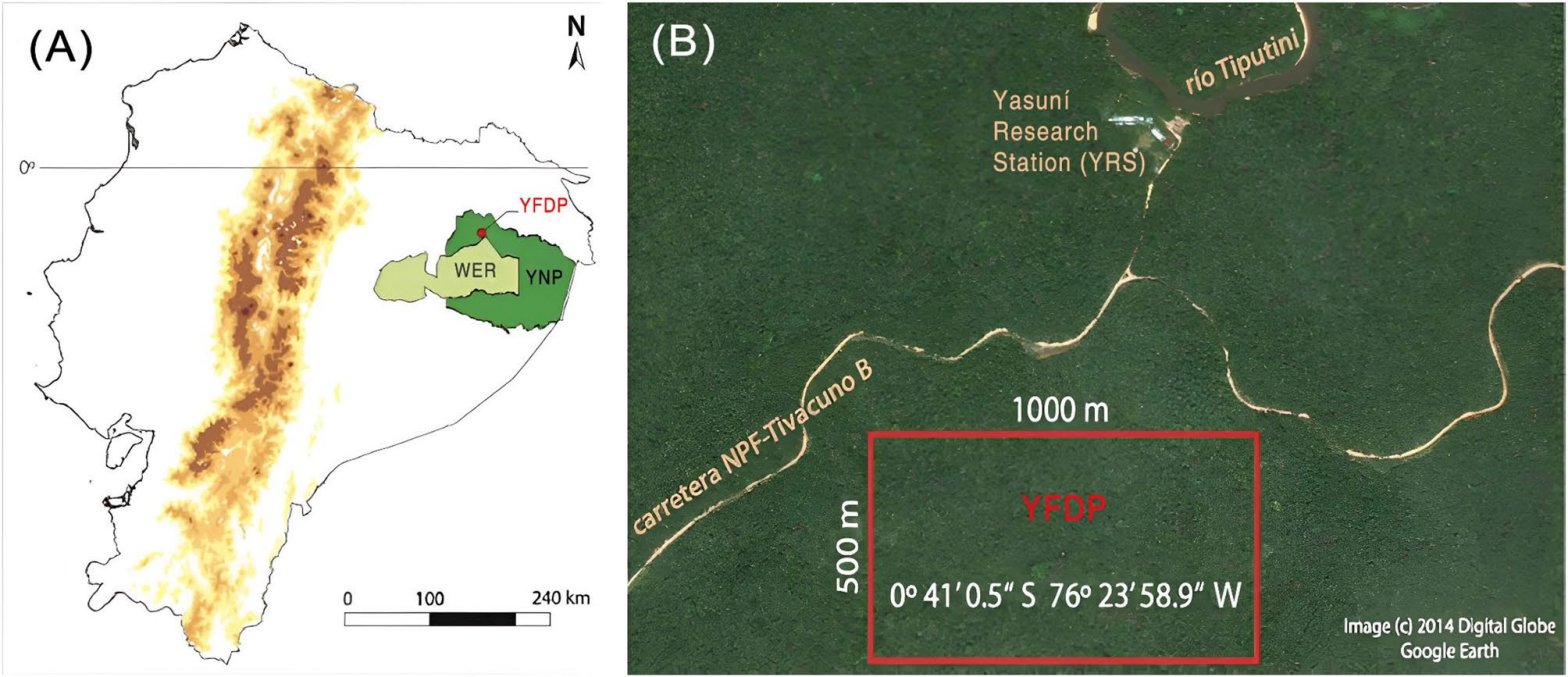
Study site location. (**A**) Ecuador continental map. Dark green areas show Yasuní National Park (YNP). The light green area shows the Waorani Ethnic Reserve (WER). The red dot marks the location of the Yasuní Forest Dynamic Plot (YFDP). (**B**) Location of the Yasuní Research Station (YRS) and the 50-ha YFDP (rectangle in red). Modified with permission from Pérez et al.^40^; Google Earth image at 800 m above the ground.

We carried out our study in the ever-wet lowland rain forest of Yasuní National Park, Ecuador (YNP; Fig. 1). The YNP is one of the most biodiverse forests in the world, with an estimated 670 species of trees in one hectare^36^. The site has an average annual precipitation of 2826 mm and a monthly temperature between 22 and 32 °C (min: 16.9 °C; máx: 38.9 °C)^37,38^. Within the park, we studied a population of *Duroia hirsuta* trees located in the long-term 50-ha “Yasuní Forest Dynamic Plot” (YFDP; 00° 41′ 0.5″ S; 076° 23′ 58.9″ W). The YFDP was established in 1995 as part of the global network of permanent plots of forest dynamics, in collaboration between the Pontifical Catholic University of Ecuador, the University of Aarhus in Denmark, and the Smithsonian Tropical Research Institute in Panamá (ForestGEO network; https://forestgeo.si.edu/sites/neotropics/yasuni) to describe the long-term demography of thousands of plant species and investigate mechanisms of coexistence. Within this plot, all trees ≥ 1 cm diameter at breast height (DBH, 1.3 m height) are labelled, mapped, and identified every five years or so^39^. The average elevation is 230 m above sea level and contains three main topographic habitats: ridge, slope or valley. Within the YFDP, a total of 1,104 morphospecies have been recorded^38^.

### *Duroia hirsuta* population and its ants

We explored the interactions among direct and indirect defence traits in the myrmecophyte *D. hirsuta*. Across its range, *D. hirsuta* hosts its strict mutualistic ant partner *M. schumanni*, but also other ants mutualistic ants such as *Azteca* spp.^14^, and other non-mutualistic ant species in various genera (*Solenopsis*, *Pheidole*, *Brachymyrmex*, *Crematogaster*, among others). In Yasuní, *Azteca* is a species-rich genus of poorly defined ants, and difficult to identify from worker castes; therefore, ants of this genus have been typified as 'spp.'. Within the 50-ha plot, 260 *D. hirsuta* individual trees were tagged and their associations with ant species were recorded yearly between 2007 and 2019. Of these, 160 trees showed continuous and monospecific presence of *M. schumanni* or *Azteca* spp. from which we selected 58 trees for analyses: *M. schumanni* (N = 29) and *Azteca* spp. (N = 29) (Fig. S1).

Originally our study design conceived plants lacking ants as a control treatment, but they were difficult to find. In fact, of the 260 *D. hirsuta* trees we surveyed, the only adult individual found alive in 2019 with no ants or no *Myrmelachista* ants recorded for more than 5 years was in dire condition, showing damaged or broken branches and a scarce number of healthy leaves.

We also acknowledge that different ant colonies of the same species may exhibit varying defence effects, a phenomenon that aligns with the concept of ‘colony personality’^41–43^. However, tracking colony turnover was beyond the scope of our field protocol, and as such, we were unable to monitor these changes directly. Despite the natural possibility of turnover among colonies of the same species, we argue that the functional basis of interspecific differences is likely to result in more pronounced effects than the intraspecific variations observed among colonies. Consequently, our analysis assumes that the comparisons made between ant species regarding their impact on host growth and defence investment remain valid, without considering the potential influence of colony turnovers within the focal trees. This approach allows us to concentrate on the differential impact of ant species, rather than the variability that might arise from changes in colony composition.

### Plants life-history trait measurements

We measured relative growth rate to evaluate the plant efficiency in the use of resources, according to the mutualistic ant species^6^. To compare the effect of each ant species against herbivores we measured herbivory as the percentage of damaged leaf area^44^. We also measured each individual’s physical defences by quantifying the shearing resistance of leaves (a measure of toughness)^12^, trichome density (a herbivore-deterring trait)^45^, and leaf dry matter content (LDMC; a key variable negatively correlated with herbivory leaf damage)^46,47^. Finally, we applied an untargeted metabolomics pipeline to quantify changes in the composition and relative abundance of plant secondary metabolites between the ant–plant mutualism partners^48,49^. We also quantified the percent of dry weight investment in soluble secondary metabolites to quantify absolute differences in chemical investment. Prior to analysis, dry weight values were converted into a 0–1 scale proportions and applied arcsine-square root transformation.

### Plant relative growth rate (RGR)

We calculated the RGR of all focal *D. hirsuta* trees. RGR was calculated using the DBH of each focal tree at *t*_1_ (2019) relative to the DBH at *t*_0_ (2007) with the following formula^14^:

$$RGR = \frac{{(DBH_{t1} /2)^{2} {-} (DBH_{t0} /2)^{2} }}{{(DBH_{t0} /2)^{2} \times \left( t \right)}},$$

where *t* is the total time in years between censuses (12 years).

### Herbivory and physical leaf-traits

#### Herbivory

Percent leaf damage by herbivores was estimated on all 58 focal *D. hirsuta* trees in November–December 2019. Standing herbivory estimates the proportion of leaf area consumed over the life span of the leaf to date, in contrast to an herbivory rate^50^. To determine leaf area loss, we took photographs of 15 randomly selected mature leaves per tree (for a total of 870 pictures) from three sections of the crown (5 leaves per section), dividing the canopy into three equal height classes. For each photograph, we used a white background with 4 dark tacks in each corner as reference points forming a 27 × 27 cm square, thus obtaining a standardised scale for the size of the leaves. To avoid tearing the leaves from the trees, we only exerted manual pressure from the base of the leaf against the white background. Using a tablet (iPad 4, model MR7C2LL/A, Apple Inc, Foxconn, Taiwan) and the *Leafbyte*® application^51^, we calculated the percentage of herbivory (cm^2^) by selecting the ‘Background Removal’ option. We compared the total area and the consumed area to calculate the percentage herbivory of each leaf.

#### Shearing resistance

Following Cárdenas et al.^52^, in 2019 we collected 10 mature leaves that showed no sign of physical damage, fungi, or galls from each focal tree. Immediately after collection, the leaves were put in wet cloth bags to maintain maximum cell turgor^46^. To analyse the mechanical resistance of leaves we used a custom steel instrument of standardised movements^52,53^. To measure shearing resistance (N × s × mm^−1^), we screwed a dual-range digital force sensor (Vernier Software & Technology, Beaverton, Oregon, EE. UU) with a peg-like folded steel sheet that supported a razor blade to the instrument [see methods and details in^52^]. *D. hirsuta* leaves were fixed between two pressure plates, leaving a space of 2 cm for shearing^48,52^. Cuts were made at different levels: two at ¼ from the base and two at ¾ of the apex of each leaf on each side, always avoiding the primary rib. The force was measured in Newtons (N) at 0.01 N precision, and the measurements were recorded as the force per unit of time (s × N; the area under the curve)^53^. The length of each cut was measured with a digital caliper to normalise the force per fracture unit N × s × mm^−1^^54^. Blades were replaced every 30 measurements to avoid their wear from influencing the result.

#### Leaf dry-matter content (LDMC)

Leaves with a high LDMC tend to be relatively harder than leaves with lower LDMC, and so provide more resistance against physical damage caused by herbivory^46,47^. LDMC is the oven-dry mass (mg) of a leaf, divided by its water-saturated fresh mass (g), expressed in mg × g^−1^^47^. To determine LDMC we used the same leaves that were used in the mechanical resistance analysis which were weighed fresh, covered with absorbent paper, dried at 60 °C for 72 h and weighed again.

#### Trichome density

We collected five young fully expanded leaves per tree and counted the trichomes in a randomly located area of 1 cm^2^ at the apex, centre, and base of each leaf using a standard stereomicroscope (Olympus Co., SZ61/SZ51, SZ2-ILST, Philippines).

### Secondary metabolites extraction

Metabolites of intermediate polarity, were extracted, separated, quantified gravimetrically, and analysed using a standard protocol for untargeted metabolomic studies^49^. For *Duroia*, these metabolites are mainly phenolics and triterpenes^55–57^. Briefly, two young expanding leaves per individual tree were collected and air-dried at room temperature with silica gel. Samples were stored at − 20 °C until processing. Following the protocol of Wiggins et al.^49^, approximately 100 mg of ground leaves was extracted with 1 ml of extraction buffer (60% acetate, 40% acetonitrile). After extraction for 10 min and centrifugation for 10 min at 13,000 rpm, the supernatant was transferred to another vial, and the process was repeated.

Extracts were diluted 1:5 with 40:60 ACN:H20 (v/v) and then analysed by C18 ultraperformance liquid chromatography coupled to mass spectrometry (UPLC-MS) using an ACQUITY UPLC I-Class system and a Waters Xevo G2 QToF mass spectrometer equipped with a LockSpray and an electrospray ionization source (Waters, Milford, MA, USA). Samples were run in negative ionization mode. LC–MS analysis was based on Wiggins et al.^49^, with some modifications, including a shorter column and run time, which we verified had sufficient chromatographic separation of peaks. We used a 22.5-min reverse-phase gradient with water (0.1% formic acid) as the mobile phase and acetonitrile (0.1% formic acid) as the stationary phase. The flow rate was 0.5 ml min, and the column temperature was 40 °C. The gradient ranged from 98 to 2% water (+ 0.1% formic acid) over the course of 16 min, followed by 6.5 min for re-equilibration (Table S1). UPLC analyses were performed at the Coley/Kursar Laboratory, University of Utah, Salt Lake City, UT, USA. The results were processed using XCMS^58–60^.

### Statistical analyses

#### Predictors of *D. hirsuta* relative growth rate

First, we examined variation in *D. hirsuta* RGR using a generalized linear mixed model (GLMM) with the ‘*glmmTMB*’ package (ver. 1.1.7;^61,62^). This statistical approach enabled us to examine the relationship between the RGR of plants and several predictor variables while accounting for the complex structure of our data. The number of conspecific plant neighbours within a 10-m radius was used as discontinuous predictor. Since we detected a residual spatial correlation in our data, as indicated by a significant Moran’s I test (I = 0.14, *p* = 0.02), we included a spatial term (longitude * latitude Cartesian coordinates) representing the plant's location within the plot (i.e., the growing site) as continuous predictor to account for the spatial structure of our data. The fixed factor in our model was ant species identity. We included habitat type (ridge, valley, slope) as a random effect, which allowed us to account for variability in RGR attributable to different habitat conditions (e.g., resource availability, enemy assemblages)^63^. We chose the gamma distribution with a log-link function since the growth rate was positive and right- skewed and did not follow a Gaussian distribution. We standardised the beta coefficients of predictor variables such as the number of neighbours and position by scaling them (subtract mean and divide by SD). We found no indication of collinearity between the covariates in the model. Second, to ensure that we were not losing valuable information from each tree sample, we tested for differences in RGR between *D. hirsuta* trees associated with *M. schumanni* and those associated with *Azteca* spp. using a non-parametric Mann–Whitney U test (Shapiro–Wilk test of normality of the residuals: W = 0.875; *p* < 0.001; although Levene’s test for homoscedasticity showed equal variances: *p* = 0.054).

#### Leaf damage and physical leaf-traits between treatments

##### Herbivory

To compare differences in leaf damage between trees with different ant mutualist, we followed the same approach as for RGR but including LDMC and shear resistance as predictors in the model. Trichome density was not included because of the small sample size. Prior to analysis, herbivory values were first converted into a 0–1 scale proportions and applied arcsine-square root transformation. Because of the nested nature of herbivory data and to make sure we were not losing valuable information from the subsampling variability we used a Friedman Test for the equality of medians (the non-parametric alternative of a Nested ANOVA; Shapiro–Wilk normality tests of residuals: W = 0.927, *p* < 0.001; Levene’s test for homoscedasticity showed equal variances: *p* = 0.903). Herbivory was analysed as a function of the type of ant and controlled for variability between leaves.

##### Shearing resistance

This measure of leaf toughness was first evaluated following the same approach as for RGR. Importantly, in the GLMM we included ant identity as a fixed factor, and the 10 m-neighbourhood and growing site (longitude * latitude Cartesian coordinates representing the plant's location within the plot) as discontinuous and continuous predictors, respectively. We also included habitat type (ridge, valley, slope) as a random effect, which allowed us to account for variability in leaf toughness attributable to different habitat conditions. As for herbivory, because of the nested nature of our sampling design, and to ensure that we were not losing valuable information due to variability in subsampling, we used the non-parametric Friedman Test for the equality of medians (Shapiro–Wilk normality tests of residuals: W = 0.335, *p* < 0.001 and Levene’s test for homoscedasticity: *p* < 0.001). Here, shearing resistance was analysed as a function of the type of ant and controlled for variability between leaves.

##### LDMC

Because of the nested nature of LDMC data and to make sure we were not losing valuable information from the subsampling variability we used a Friedman Test for the equality of medians. LDMC was analysed as a function of the type of ant and controlled for variability between leaves to test for differences in LDMC between *D. hirsuta* trees with *M. schumanni* or *Azteca* spp (Shapiro–Wilk normality test of residuals: W = 0.589, *p* < 0.001; Levene’s test for homoscedasticity: *p* < 0.001).

##### Trichome density

We performed a nested ANOVA to compare the average number of trichomes present in the leaves of five random *D. hirsuta* focal trees (one leaf per tree) hosting each of the two ant species (N = 10), with trichome density as the response variable, and the part of the leaf (i.e., apex, centre, base) nested within each ant species (n = 15; *M. schumanni* or *Azteca* spp.). This was performed to account for the potential bias of not independent subsamples within replicates. Prior to any statistical test, trichomes counts were square-root transformed; residuals showed to be normally distributed (Shapiro–Wilk normality test: W = 0.973, *p* = 0.611; and had equal variances: Levene’s test for homoscedasticity: *p* = 0.567).

### Chemical leaf-trait analyses

We used a compound-based molecular networking approach, where we first grouped related features into compounds and then generated (a) an individual-by-compound abundance matrix and (b) a compound-by-compound MS/MS cosine similarity matrix. These data were combined into a pairwise species similarity matrix, which accounts for both shared compounds between individuals and the MS/MS structural similarity of unshared compounds^64^. This similarity matrix of relative abundances of UPLC-MS metabolites was used to make a hierarchical cluster (to determine the similarity of the samples in a dendrogram according to metabolite presence-absence, relative abundance and chemical structure), principal coordinates analysis (PCoA) (to observe the chemical similarity between trees with different ant species), and a permutational multivariate analysis of variance (PERMANOVA) to test the interaction effect of the type of ant and the DBH difference over 12 years and whether they have an effect on the relative abundances of metabolites. Hierarchical clustering was performed in R v4.2.2^65^ with the “pvclust” package^66^. PCoA was carried out in PAST v4.04 software^67^. PERMANOVA was performed in XLSTAT^68^. For this analysis the age effect (i.e., a sapling grows faster than an adult tree that is reaching its maximum size;^68^) in the DBH of the trees was eliminated by calculating the quartiles and their mean with the DBH differences. For this, we used the following formula:

$$x=\frac{log(DBH \,difference+1) }{log(quartile\, mean+1)}$$

The values of dry weight invested in the production of secondary metabolites were tested using a Mann Whitney U test (Shapiro–Wilk test: W = 0.848, *p* < 0.001; Levene’s test for homoscedasticity: *p* = 0.346).

### Survival exploration of trees in the long-term presence/absence of ants

The unexpected discovery that only one individual living plant remained without the presence of ants for no more than 4 years, as per our sampling design, drew our attention. For this reason we could not have a control group and led us to analyse the survival percentage of *D. hirsuta* trees at the entire 50-ha plot. For this we divided the total number of target trees (N = 260) into 4 categories: (A) colonised by either *M. schumanni* or *Azteca* spp. only, called 'monospecific colonisation' (n = 164); (B) with ants absent for ≥ 5 consecutive years or no ant colonisation at all (n = 28); (C) trees that had some form of ant colonisation for only 4 years or less, either monospecific or heterospecific (n = 16); and (D) trees that had heterospecific colonisation, i.e., where we found either two ant species at the same time or witnessed colony exchange during tree individuals’ lifespan (n = 52). We used our 12-year census of the population of *D. hirsuta* individuals in the 50-ha YFDP to assess the percentage of alive plants in the 4 categories.

### Plant material

Voucher specimen of *Duroia hirsuta* (Poepp. & Endl.) K.Schum., 1888 (Rubiaceae) (#236695) is deposited at QCA Herbarium, Pontificia Universidad Católica del Ecuador; identified by Álvaro J. Pérez. Collection of plant material complied with institutional, national, and international guidelines and legislation. Ministerio de Ambiente del Ecuador Permit number: 012-2014-FLO-MAE-DPAO-PNY.

## Results

### Life-history traits of evaluated trees

The relative growth rate (RGR), herbivory, shearing resistance, leaf dry matter content (LDMC), and trichome density of *D. hirsuta* associated with *M. schumanni* and *Azteca* spp. trees were individually analysed using both parametric and non-parametric tests on non-averaged data. These individual evaluations aim to take into account the variability within groups and ensure that important information is not lost in evaluations that use average values, such as the GLMM.

#### RGR

**Figure 2:**
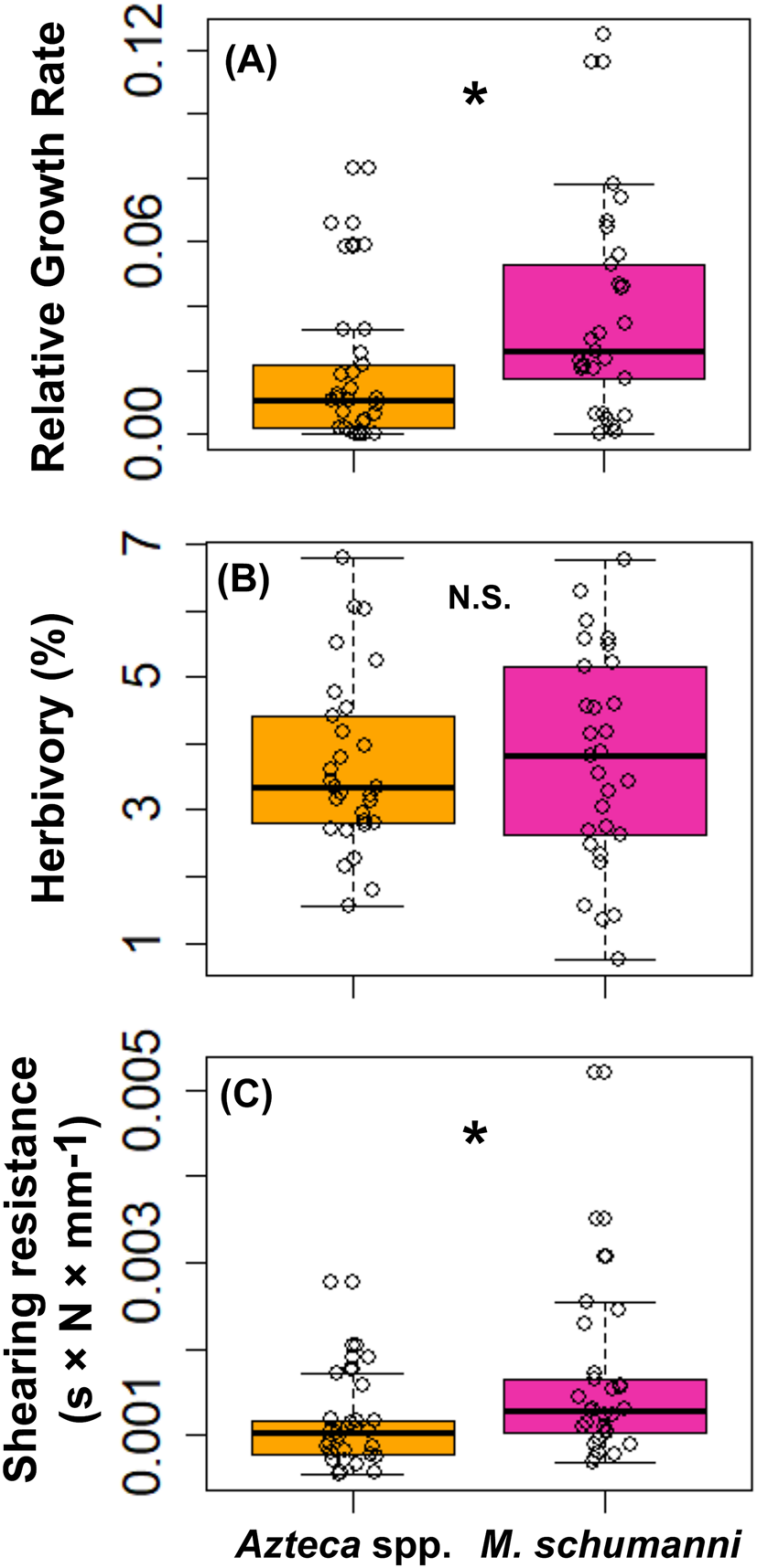
Box plots and stripcharts of *D. hirsuta* traits in mutualism with *Azteca* spp. and *M. schumanni*. (**A**) shows the trees relative growth rates between 2007 and 2019; (**B**) the percentage of leaf damage by herbivory; (**C**) the leaf shearing resistance. * Indicates a significance of *p* < 0.05; N.S. means non-significance with *p* > 0.05. Results of GLMM statistical analyses are shown in Table 1.

Individual *D. hirsuta* trees hosting *M. schumanni* had almost double the RGR (mean ± SD = 0.037 ± 0.03) compared to those hosting *Azteca* spp. (0.018 ± 0.02, Fig. 2A) (Mann–Whitney U test: U = 247; *p* = 0.007).

#### Herbivory

The average area of all the measured leaves per tree was 90.01 cm^2^ ± 46.64 (mean ± SD). The average consumed area was 7.10 cm^2^ ± 9.44 (i.e., 8.86%) per leaf. *D. hirsuta* trees in mutualism with *M. schumanni* had similar leaf area (92.51 cm^2^ ± 52.80) and levels of herbivory (7.17 cm^2^ ± 9.36; 9.04%) compared to trees in mutualism with *Azteca* spp. (area: 87.66 cm^2^ ± 39.39; herbivory: 7.04 cm^2^ ± 9.52, 8.68%) (Friedman Test for herbivory: *d.f.* = 1; *χ*^2^ = 0.067, *p* = 0.796; Fig. 2B).

#### Shearing resistance

Individuals hosting *M. schumanni* showed significantly higher leaf shearing resistance (0.0017 s × N/mm ± 0.001) compared to trees hosting *Azteca* spp. (0.0011 s × N/mm ± 0.0005) (Friedman Test: *d.f.* = 1; *χ*^2^ = 10, *p* = 0.0016; Fig. 2C).

#### LDMC

We found marginally significant differences between the LDMC from trees hosting *M. schumanni* and *Azteca* spp.: average (± SD) of 0.337 mg × g^-1^ (± 0.06) and 0.329 mg × g^-1^ (± 0.12), respectively (Friedman Test: *d.f.* = 1; *χ*^2^ = 3.6, *p* = 0.058).

#### Trichome density

We found no significant difference in the trichome density from different parts of the leaf on *D. hirsuta* trees with *M. schumanni* (17 × cm^-2^ ± 11) compared to *D. hirsuta* trees with *Azteca* spp. (12 × cm^-2^ ± 7) (nested ANOVA: *d.f.* = 1, F = 2.434, *p* = 0.132).

### Plant traits relationship to ant identity and the growing site

Because tree performance and traits can also vary in response to the local neighbourhood, we included the number of conspecifics within a 10-m radius of each focal tree in more complex models of *D. hirsuta* performance and traits.

In *D. hirsuta* trees, differences among trees harbouring different ant species in RGR and shearing resistance were predicted by ant identity (Table 1; Fig. 2A–C) and the growing site (Table 1). Trees hosting *M. schumanni* grew faster compared to trees hosting *Azteca* spp. from 2007 to 2019 (*χ*^2^ = 9.140, *p* = 0.003). The growing site also predicts higher RGRs of *D. hirsuta* in this forest (Z = 2.878, *p* = 0.004), while the 10 m-neighbourhood showed to have nor positive or negative effect (Z = -1.020, *p* = 0.308). There were no differences in leaf herbivory and there was no association to any potential predictors. Trees hosting *M. schumanni* produced more resistant leaves than those hosting *Azteca* spp. (*χ*^2^ = 7.299, *p* = 0.007) and this has shown to also be associated to the growing site (Z = 2.060, *p* = 0.039).

**Table 1:** Generalised linear mixed models (GLMM) analysing the effects of ant identity, conspecific neighbourhood, and growing site on (A) plants’ relative growth rate (RGR; between 2007 and 2019), (B) herbivory and (C) leaf shearing resistance in 2019.

Effect	d.f	Estimate	*χ*^2^	*Z*	*p*
(A)					
Growth-rate					
Ant identity	1	0.020	9.140	3.023	**0.003**
10 m-neighbourhood	1	− 0.003		− 1.020	0.308
Growing site	1	0.009		2.878	**0.004**
(B)					
Herbivory					
Ant identity	1	− 0.009	0.007	− 0.081	0.935
LDMC	1	− 0.008		− 0.150	0.881
Shearing resistance	1	0.072		1.171	0.242
10 m-neighbourhood	1	0.019		0.334	0.738
Growing site	1	0.080		1.468	0.142
(C)					
Shearing resistance					
Ant identity	1	− 0.156	7.299	− 2.700	**0.007**
10 m-neighbourhood	1	0.074		1.250	0.211
Growing site	1	0.125		2.060	**0.039**

In both (A) and (C), models were controlled by type of habitat (ridge, slope, valley). For neighbourhood effects we first conducted pairwise Spearman correlation analyses on neighbour counts at radii of 5, 10, 15, and 20 m. In all cases correlations were significant (Table S2), hence we chose for 10 m as an intermediate value to incorporate into the model. In (B), leaf herbivory model included leaf dry matter content (LDMC) and shearing resistance as predictor variables. The *χ*^2^ and *p* values of fixed factors were estimated using type II Wald tests and random effects were estimated using likelihood-ratio tests with one degree of freedom. Significant *p* values are in bold. Full raw data for GLMM models is available in Table S3.

### Similar chemical leaf profiles of trees living in and out devil’s gardens

**Figure 3:**
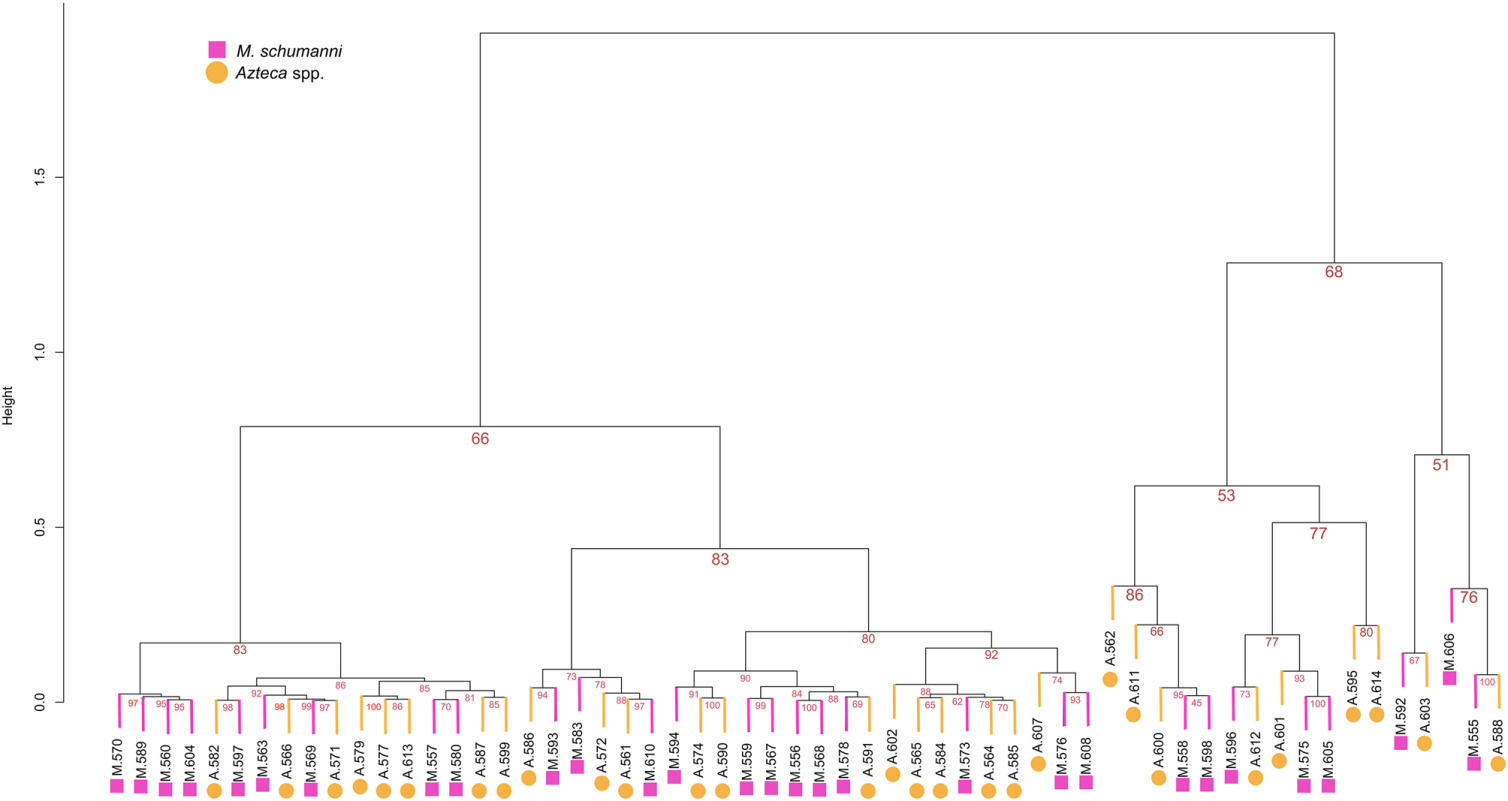
Secondary metabolites dendrogram of *D. hirsuta* associated with *M. schumanni* and *Azteca* spp. The dendrogram is based on the similarity matrix of relative abundances of UPLC-MS metabolites and shows that both treatments are chemically similar because trees with *M. schumanni* and *Azteca* spp. are mixed within the clusters. Red numbers represent the Approximately Unbiased (AU) *ps*; these indicate the probability that the samples below that point are a cluster. Clusters with values of 95 mean *p* = 0.05, indicating that these clusters are strongly supported by the data.

**Figure 4:**
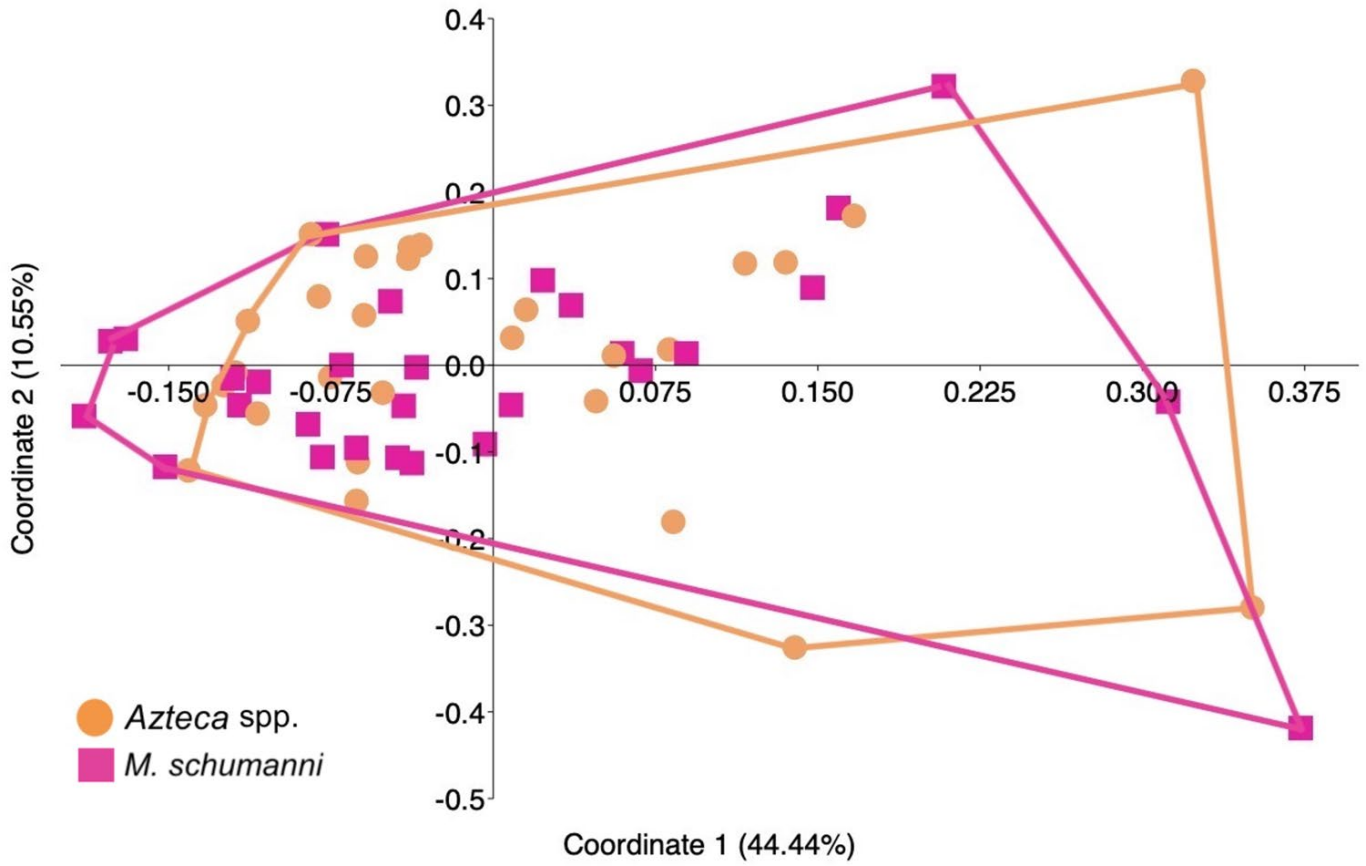
Principal coordinate analysis (PCoA) of the chemical similarity matrix. Individuals of *D. hirsuta* in mutualism with *M. schumanni* (pink squares) and *Azteca* spp. (orange dots) show an overlap in the PCoA graph made from the chemical similarity matrix.

The profiles of defensive metabolites of trees associated with either *M. schumanni* or *Azteca* spp. did not show significant differences. Figures 3 and 4 show that the metabolites composition of the leaf of trees may depend on other factors than the hosting specificity of symbiotic ants. The PERMANOVA showed that the ant identity (*d.f.* = 1, *F* = 0.996, *p* = 0.410), DBH (*d.f.* = 1, *F* = 0.733, *p* = 0.713), and the interaction between both variables (*d.f.* = 1*F* = 1.235, *p* = 0.231) did not have significant effects on the quantity of the produced secondary metabolites between trees living in and out devil’s gardens. The values of dry weight invested in the production of secondary metabolites showed no differences between trees hosting *M. schumanni* or *Azteca* spp. (Mann–Whitney U = 269, *p* = 0.147).

### *Duroia hirsuta* survival in the presence or absence of ants

During our 12-year survey of 260 *D. hirsuta* trees, 164 showed monospecific continuous/iterative recolonization of only *M. schumanni* or *Azteca* spp. ants. Of these, 115 (70.1%) hosted *M. schumanni* (Table 2A; Table S4A). These trees with a continuous ant presence survived well: 98.3% of these 164 trees were alive (Table 2; Table S4A). On the other hand, only one of the 28 individuals recorded with absence of ant species for ≥ 5 years was found alive by 2019 (3.6% survival; Table 2B; Table S4B). Similarly, of the 16 trees where we recorded ants presence for ≤ 4 years, only one was found alive in 2019 (6.25% survivorship; Table 2C; Table S4B).

**Table 2:** Survivorship of *Duroia hirsuta* trees in Yasuní Forest Dynamic Plot, Ecuador in the long-term presence/absence of ant species.

Ant groups	(A) Monospecific colonisation (*n* = 164)	(B) Total absence or ≥ 5 years of no colonisation (*n* = 28)	(C) ≤ 4 years of mono- or heterospecific colonisation (*n* = 16)	(D) Heterospecific colonisation (*n* = 52)
*Myrmelachista*	115	–	9	16
*Azteca*	48	–	7	25
*Crematogaster*	–	–	1	23
*Brachymyrmex*	–	–	1	16
*Solenopsis*	–	1*	1	15
*Wasmannia*	–	–	0	9
*Pheidole*	–	–	1	7
*Pseudomyrmex*	–	–	1	6
*Pachycondyla*	–	–	0	5
*Monomorium*	–	–	0	3
*Camponotus*	–	–	0	2
*Cephalotes*	–	–	0	2
*Dolichoderus*	–	–	0	1
*Nylanderia*	–	–	0	1
No Ant	–	28	–	17
**Alive**	**98.8%**	**3.6%**	**5.9%**	**98.1%**
**Dead**	**1.2%**	**96.4%**	**94.1%**	**1.9%**

Table is divided in four categories: (A) ‘Monospecific colonisation’ when trees have shown the presence of one species only of *Myrmelachista schumanni* or *Azteca* spp. during our survey; (B) ‘No colonisation’ when trees have shown ≥ 5 years or total absence of ants through the years and were found still alive; (C) Those trees showing one up to 3 years (4 years for one tree only) of mono- or heterospecific colonization; (D) ‘Heterospecific colonisation’ when trees have shown the presence of at least two species (this includes absence of foraging ants in time intervals).

*n* means *Duroia* trees abundance. “–” means no ants found. *database record of the presence of an ant species but lacking any other ant colonisation for ≥ 5 years. Alive and dead trees by 2019 are shown in bold at the bottom in terms of percentage survival.

Similar to trees with continuous monospecific ant presence, trees with heterospecific colonisation of ants (*n* = 52) had high survival (only one individual found dead by 2019) (Table 2D; Table S5). Of these trees, we found *Azteca* as the most common throughout the years (19.1% of occurrence), followed by *Crematogaster* (17.6%), *Brachymyrmex* (12.2%), *Myrmelachista* (12.2%) and *Solenopsis* (11.5%) (72.5% of all occupations; Table 2D).

## Discussion

Our 12-year survey of *D. hirsuta* and its principal mutualist ants (*M. schumanni* and *Azteca* spp.) showed that their presence in at least one moment during its lifetime (both monospecific and heterospecific colonisation) greatly improved *D. hirsuta* survival: > 98% survival with ≥ 5 years with symbiotic ants; but only ~ 6% survival with ≤ 4 years with symbiotic ants; and only 3.6% survival with no ants ever recorded. Moreover, we found that 38%–70% of the sampled trees hosted *M. schumanni* during their entire life (compared to 22%–29% for *Azteca* spp.), suggesting that *D. hirsuta* is more often found inside gardens than outside gardens and that the benefits of hosting *M. schumanni* may outweigh any negative effects of high conspecific density. Within the 25-ha of the YFDP, based on the census data from 2007–2017, *D. hirsuta* showed an average annual mortality rate of 1.97% compared to the remaining plant species of the plot, which was 2.74% ± 0.196 (mean ± 95% confidence interval; rates calculated following the ForestGEO network protocol at https://forestgeo.si.edu/protocols/tree-mortality-damage-protocol). However, all except one individual of *D. hirsuta* trees censused without ant mutualists were found dead, and 98.8% of the trees censused in mutualism with *M. schumanni* or *Azteca* spp. were found alive. These results suggest that in Amazonian forests, indirect defences through association with ants may be mandatory over the long term, since survival seems not possible for this tree species without any ant mutualism^6^.

Considering the role of myrmecophytic mutualism in the diameter growth of *D. hirsuta*, our results (Fig. S2A) are similar to those of Báez et al.^14^ conducted in the same plot, who found a growth effect on smaller but not on larger trees over both the short and long term (6 and 18 years), where 56% of *D. hirsuta* trees hosted *M. schumanni* and the remaining 44% of trees hosted other ant species. Furthermore, Frederickson and Gordon^69^ also demonstrated significantly faster growth of *D. hirsuta* hosting *M. schumanni* compared to those with *Azteca* spp. or other ant species. In our 12-year survey, we showed that mutualism with ants was found to be beneficial, since trees that were associated exclusively with *M. schumanni* grew twice as fast as trees hosting *Azteca* spp. Our results also show that growth was not consistent over the 12 years study. For example, for *D. hirsuta* with *M. schumanni*, growth was faster during the first 5 years than the following 7 years (Fig. S2B). The dynamics of the mutualism may evolve as the plant progresses through different ontogenetic stages and the growth of *D. hirsuta* may be determined by variation in the direction and strength of the mutualist relation with the ant, tree size (negative effect), and the density of conspecific neighbours (positive effect)^14^. Moreover, CNDD did not affect *D. hirsuta* performance for small trees (but less consistent on large ones), most likely because the presence of ants reduces the impact of density dependent stressors such as insect herbivores or fungal pathogens^14,70–72^. This is, however, debatable since *Myrmelachista* in mutualism with *D. hirsuta* trees seem to not attack potential enemies, in contrast to *Myrmelachista* associated with other tree species where they are more aggressive^73^. Alternatively, the increased availability of resources associated with devil’s gardens is a more conceivable mechanism driving increased growth and survival of *D. hirsuta* because we know that the main protection in the *Myrmelachista*-*Duroia* system is the clearing of plants from the host’s surroundings^28,30,74^. The increased light and/or water availability and nutrient input from ant mutualists could decrease resource limitation of herbivores (i.e., better quality of leaves)^75^, but also may allow *D. hirsuta* to invest in its own direct defences and to better cope with herbivore damage events when outbreaks occur.

Leaf toughness is the most general physical defence that can be compared among plant taxa found within tropical forests^52,76–78^. Leaf toughness is expected to prevent herbivory and thus reduce mortality due to physical damage^79,80^. Therefore, increased leaf toughness would be expected to increase the leaf lifespan and overall survival of the whole individual, especially in shade-tolerant species and individuals^81^. Structural traits can prevent the action of predators, particularly chewing invertebrates (considered the dominant herbivore guild in tropical rainforests) as they would avoid feeding on hard vein tissues^76,82^. Leaf physical resistance to damage has also been shown to be negatively correlated with herbivory and positively correlated with leaf lifespan, seedling survival, and shade tolerance in 19 species in the Bolivian Amazon^81^. However, our results (greater shearing resistance in *D. hirsuta* with *M. schumanni* than with *Azteca* spp.) suggest that leaf toughness might vary in response to mutualist identity. One possible mechanism is that the higher resource availability around *D. hirsuta* individuals in association with *M. schumanni* allows them to invest more resources in tougher (i.e., more shear-resistant) leaves. This is in contrast to other leaf morphological traits -marginal difference in LDMC, and no difference in trichome density-, between trees with the two ant mutualists, and needs further evaluation. Also, the percentage of dry weight for the production of secondary metabolites was similar for trees harbouring different ant species. Although we observed caterpillars of *Adelpha* spp. Nymphalidae (Lepidoptera) and adults of Chrysomelidae (Coleoptera) feeding on young and mature leaves on some *D. hirsuta* (PA, pers. obs.) herbivory was not significantly different between trees hosting *M. schumanni* or *Azteca* spp., and may be largely due to the general effectiveness of the plant's direct defences.

At the chemical level, there were no significant differences in leaf secondary metabolite composition between *D. hirsuta* trees associated with *M. schumanni* and those in mutualism with *Azteca* spp. One possible explanation for this lack of differences in chemistry is the role of secondary metabolites in *D. hirsuta* as constitutive defences. Even when an ant colony is present on a myrmecophyte it may not provide effective defence against herbivores^83^ (see also Cárdenas^9^), so myrmecophytic plants may benefit from having other resistance traits when indirect defence fails. Although inducible defences might have greater advantages in temperate than tropical regions because they are less costly, selection may favour constitutive defences in tropical plants because herbivore pressure is higher and constant throughout the year^83,84^.

Bixenmann et al.^84^ argued that species adapted to environments with high levels of herbivory will favour constitutive defences rather than induced defences. This can reinforce our principal idea that secondary metabolites function as a constitutive defence in our study, given that we only performed biochemical analysis in expanding leaves. In addition, Frederickson et al.^83^ stated that *Cordia nodosa* (Boraginaceae) presented greater values of leaf toughness with more trichomes and more total phenolics (direct defences) when ants were absent than in the presence of ants. In our study we were not able to perform a comparison between trees with and without ants because of the level of deterioration and lack of healthy leaves shown by the only tree found alive without ants for more than 5 continuous years. Perhaps *D. hirsuta* trees without ants are capable of inducing secondary metabolites, but that is not enough for their survival, showing the importance of their mutualism with ants (regardless of the species they are associated with). It is said that biotic defence, in the form of ant mutualists, is the most effective protection against herbivory, which corroborates numerous studies showing that ants often tightly coevolved defence^85^. Moreover, Müller et al.^21^ hypothesised that plants colonised by ants do not require inducible direct defence mechanisms but did find that insect feeding led to increases in jasmonate levels and the emission of herbivore-induced plant volatiles (HIPVs) regardless of the status of colonisation. This suggests that perhaps ant protection is not sufficiently reliable under all conditions.

As well as whole-plant ontogeny and its influence on the production of secondary metabolites, leaf ontogeny is also important. We only analysed expanding leaves; thus, it would be important to determine if mature leaves had more or less secondary metabolites than expanding leaves. Expanding leaves of most species experience considerably higher rates of herbivory than mature leaves^86^. In tropical forests, young leaves of some plant groups exhibit an even greater diversity of defences than mature leaves because they can experience herbivore pressure throughout the year, in contrast to temperate forests where they can rely on early spring flushing to escape herbivores^86,87^. Expanding leaves can have higher concentrations of secondary metabolites than mature leaves, as well as compounds not found in the older leaves^87^. There is also evidence that, in general, immature and unexpanded leaves have higher nutrient concentrations and higher secondary metabolite concentrations^49^, whereas fully expanded mature leaves are tougher and have higher levels of indirect defences^88^. Hence, leaf ontogeny, rather than any mutualist, may be driving the similarity in secondary metabolites between trees with different ant species. We need to confirm this idea with information on secondary metabolites from mature leaves.

### What is the importance of studying the impact of 12 consecutive years of monospecific mutualism between *D. hirsuta* and its hosts?

A strong component of our study design was to consider length of time that different ant species (*M. schumanni*, *Azteca* spp. and others) lived in mutualism with one individual plant. This is the first time, to our knowledge, that research on the ecology of *D. hirsuta*’s natural history considers such long-term strict interactions, portraying the strong dependency between the plant and its ants. Because of the unique behaviour of *M. schumanni*, and that *D. hirsuta* can be associated with other ants for long periods of time (i.e., our results showed that some trees have never hosted *M. schumanni* over the 12-years study), we were able to take advantage of this natural experiment. On one hand, the ant *M. schumanni* is the only species to nest in the domatia, clears the surrounding area and reduces competition, and may probably increase the availability of resources such as water, light and/or nutrients. In Yasuní myrmecophyte saplings (including *D. hirsuta*) produce domatia independently of the presence of ants when they typically reach ~ 1 cm of DBH (PA pers. obs.). On the other hand, other mutualistic ants such as *Azteca* spp. nest in nearby heterospecific trees and do not isolate the tree from the rest of the forest vegetation (Fig. S1), but may also bring benefits to the host plant. Long-term studies of this type of strict monospecific mutualism between ants and their host trees can offer deep insights into their impact on the structure of ecological systems, explain the importance of symbiotic relationships that maintain ecological stability, and how these interactions shape community dynamics and individuals' resource allocation and productivity. These studies help enhance our understanding of the factors behind the ecological stability and resilience of such systems.

Since mutualist relationships significantly influence the composition, structure, function, and dynamics of plant communities, it is vital to better understand these relatively long processes affecting the community through individuals. For example, myrmecophyte protection can extend beyond the focal tree where (1) smaller trees that host mutualist ants consistently exhibit faster growth when surrounded by a greater number of neighbouring trees^14^, and (2) trees growing next to plants with extrafloral nectaries have more ants and fewer harmful caterpillars compared to other trees of the same species located farther away, also showing different defence traits and elevated growth rates likely because they do not need to invest and allocate as many resources to protect against herbivores and pathogens^15^. Mutualisms can also impact resource allocation and productivity, as ant-tree interactions can lead to more efficient nutrient cycling and energy flows within the ecosystem. This, in turn, can enhance the productivity of the ecosystem. Such effects illustrate the vital role mutualistic relationships play in maintaining ecological balance and supporting diverse life forms, and can be known only from long-term surveys.

To further understand whether the ant partner in these mutualisms benefits the plant in a way that translates into better growth or reproduction (and ideally fitness), finer analyses and experiments must be carried out taking advantage the nature of the different types of mutualisms. Studies should consider a larger number of samples when possible, and diversify the surveyed variables. Some examples are: (i) the effect of ants species presence on plant ontogeny; (ii) qualitative and quantitative analyses of variation in resource availability (e.g., water, light, macro- and micronutrients); (iii) document allocation of macro nutrients such as N using stable isotopes tracers (it is likely that plants capable of utilising insect waste deposits will gain a competitive advantage^16^); (iv) describe the soil microbiota (fungi and bacteria) diversity and structure associated with the target trees; and (v) quantify soil respiration at the root level in and outside devil’s gardens. In all cases we suggest also trying to distinguish any differences in the ant–plant mutualism between male and female individuals of dioecious populations (of which *D. hirsuta* is one) (i.e., females have a greater resource allocation for reproduction that becomes evident when *Myrmelachista* ants are present^89,90^), and consider any alteration in colony dynamics (such as shifts in the use of resources or colony turnover of the same ant species). Similarly, a better understanding of the wide variety of direct and indirect defences that can be either constitutive or induced, and how these strategies can be influenced by plant and leaf ontogeny, herbivore pressure, type and quantity of volatile organic compound liberated after herbivore attack (and if different ant species respond to “the call for help” and how), conspecific negative density dependence effect, among other factors. An understanding of these factors and how they interact with each other is important to better comprehend the historical eco- co-evolutionary ant-plant mutualistic systems.

## Conclusions

Our study reaffirms the unique Devil’s garden mutualistic system as an exemplary model for exploring intricate plant–animal interactions. This system not only provides insight into the co-evolutionary adaptations necessary for survival in challenging environments but also enhances our understanding of ecological diversification and species coexistence. The variation in life-history traits linked to different ant partners illustrates how mutualisms can influence microevolutionary processes and community structure. By examining these dynamics, our research contributes to a deeper understanding of how mutualistic interactions can shape ecological communities and drive evolutionary change. This work encourages further exploration into the mechanisms by which mutualisms influence ecological and evolutionary outcomes, potentially offering new perspectives on the resilience and adaptability of ecosystems in the face of environmental stressors.

### Supplementary Information

Supplementary Information.

## Data Availability

All data generated or analysed during this study are included in this published article in Supplementary Materials.

## References

[1] Nelsen, M. P., Ree, R. H. & Moreau, C. S. Ant–plant interactions evolved through increasing interdependence. *Proc. Natl. Acad. Sci.* **115**, 12253 (2018). 30420513 10.1073/pnas.1719794115 PMC6275543

[2] Wilson, E. O. & Hölldobler, B. The rise of the ants: a phylogenetic and ecological explanation. *Proc. Natl. Acad. Sci.* **102**(21), 7411–7414 (2005). 15899976 10.1073/pnas.0502264102 PMC1140440

[3] Parker, J. & Kronauer, D. J. How ants shape biodiversity. *Curr. Biol.* **31**(19), R1208–R1214 (2021). 34637733 10.1016/j.cub.2021.08.015

[4] Shingleton, A. W., Stern, D. L. & Foster, W. A. The origin of a mutualism: a morphological trait promoting the evolution of ant-aphid mutualisms. *Evolution* **59**, 921 (2005). 15926702 PMC2965734

[5] Six, D. L. Climate change and mutualism. *Nat. Rev. Microbiol.* **7**, 686 (2009). 19756007 10.1038/nrmicro2232

[6] Frederickson, M. E. Ant species confer different partner benefits on two neotropical myrmecophytes. *Oecologia* **143**, 387 (2005). 15711821 10.1007/s00442-004-1817-7

[7] Mayer, V. E., Frederickson, M. E., McKey, D. & Blatrix, R. Current issues in the evolutionary ecology of ant–plant symbioses. *New Phytol.* **202**, 749 (2014). 24444030 10.1111/nph.12690

[8] Rosumek, F. B., Silveira, F. A. O., de S. Neves, F., de U. Barbosa, N. P., Diniz, L., Oki, Y., et al. Ants on plants: a meta-analysis of the role of ants as plant biotic defenses. *Oecologia*, **160**, 537 (2009). 10.1007/s00442-009-1309-x 19271242

[9] Cárdenas, R. E. Casual encounter?. *Front. Ecol. Environ.* **17**, 390 (2019). 10.1002/fee.2095

[10] González-Teuber, M. & Heil, M. Comparative anatomy and physiology of myrmecophytes: ecological and evolutionary perspectives. *Res. Rep. Biodiversity Stud.* **4**, 21 (2015).

[11] Heil, M. & McKey, D. Protective ant-plant interactions as model systems in ecological and evolutionary research. *Ann. Rev. Ecol. Evol. Syst.* **34**, 425 (2003). 10.1146/annurev.ecolsys.34.011802.132410

[12] Orona-Tamayo, D. & Heil, M. Stabilizing mutualisms threatened by exploiters: new insights from ant–plant research. *Biotropica* **45**, 654 (2013). 10.1111/btp.12059

[13] Chamberlain, S. A. & Holland, J. N. Quantitative synthesis of context dependency in ant–plant protection mutualisms. *Ecology* **90**, 2384 (2009). 19769117 10.1890/08-1490.1

[14] Báez, S. *et al.* Ant mutualism increases long-term growth and survival of a common Amazonian tree. *Am. Nat.* **188**, 567 (2016). 27788348 10.1086/688401

[15] Staab, M. *et al.* Dear neighbor: Trees with extrafloral nectaries facilitate defense and growth of adjacent undefended trees. *Ecology* **104**, e4057 (2023). 37078562 10.1002/ecy.4057

[16] Sagers, C. L. Nutrient acquisition and concentration by ant symbionts: the incidence and importance of biological interactions to plant nutrition. In *Ecological Aspects of Nitrogen Metabolism in Plants* (eds Polacco, J. C. & Todd, C. D.) 715–765 (Wiley, 2011). ISBN:9780813816494

[17] Thompson, J. N. Reversed animal–plant interactions: the evolution of insectivorous and ant–fed plants. *Biol. J. Linnean Soc.* **16**(2), 147–155 (1981). 10.1111/j.1095-8312.1981.tb01647.x

[18] Wagner, D. The influence of ant nests on *Acacia* seed production, herbivory and soil nutrients. *J. Ecol.* **85**, 83–93 (1997). 10.2307/2960629

[19] Fischer, R. C., Wanek, W., Richter, A. & Mayer, V. Do ants feed plants? A 15N labelling study of nitrogen fluxes from ants to plants in the mutualism of Pheidole and Piper. *J. Ecol.* **91**, 126 (2003). 10.1046/j.1365-2745.2003.00747.x

[20] Gegenbauer, C., Mayer, V. E., Zotz, G. & Richter, A. Uptake of ant-derived nitrogen in the myrmecophytic orchid *Caularthron bilamellatum*. *Ann. Bot.* **110**, 757 (2012). 22778148 10.1093/aob/mcs140 PMC3423799

[21] Müller, A. T. *et al.* Combined -omics framework reveals how ant symbionts benefit the Neotropical ant-plant *Tococa quadrialata* at different levels. *iScience* **25**, 105261 (2022). 36274949 10.1016/j.isci.2022.105261 PMC9579026

[22] Sagers, C. L., Ginger, S. M. & Evans, R. D. Carbon and nitrogen isotopes trace nutrient exchange in an ant-plant mutualism. *Oecologia* **123**, 582 (2000). 28308767 10.1007/PL00008863

[23] McNett, K. *et al.* Stable isotope investigation of a cryptic ant-plant association: Myrmelachista flavocotea (Hymenoptera, Formicidae) and Ocotea spp. (Lauraceae). *Insectes Sociaux* **57**, 67–72 (2010). 10.1007/s00040-009-0051-z

[24] Wagner, D. & Fleur-Nicklen, E. Ant nest location, soil nutrients and nutrient uptake by ant-associated plants: Does extrafloral nectar attract ant nests and thereby enhance plant nutrition?. *J. Ecol.* **98**(3), 614–624 (2010). 10.1111/j.1365-2745.2010.01640.x

[25] Dejean, A. *et al.* Predation success by a plant–ant indirectly favours the growth and fitness of its host myrmecophyte. *PLoS ONE* **8**(3), e59405 (2013). 23516632 10.1371/journal.pone.0059405 PMC3597600

[26] Yamawo, A. & Hada, Y. Effects of light on direct and indirect defences against herbivores of young plants of *Mallotus japonicus* demonstrate a trade–off between two indirect defence traits. *Annals of Botany* **106**(1), 143–148 (2010). 20472698 10.1093/aob/mcq093 PMC2889801

[27] van Velzen, E. & Etienne, R. S. The importance of ecological costs for the evolution of plant defense against herbivory. *J. Theor. Biol.* **372**, 89–99 (2015). 25747775 10.1016/j.jtbi.2015.02.027

[28] Frederickson, M. E., Greene, M. J. & Gordon, D. M. ‘Devil’s gardens’ bedevilled by ants. *Nature* **437**(7058), 495 (2005). 16177778 10.1038/437495a

[29] Zook, D. Tropical rainforests as dynamic symbiospheres of life. *Symbiosis* **51**, 27–36 (2010). 10.1007/s13199-010-0071-5

[30] Davidson, D. W. & McKey, D. Ant-plant symbioses: Stalking the Chuyachaqui. *Trends Ecol. Evol.* **8**, 326 (1993). 21236183 10.1016/0169-5347(93)90240-P

[31] Frederickson, M. E. & Gordon, D. M. The devil to pay: a cost of mutualism with *Myrmelachista schumanni* ants in ‘devil’s gardens’ is increased herbivory on *Duroia hirsuta* trees. *Proc. R. Soc. B* **274**(1613), 1117 (2007). 17301016 10.1098/rspb.2006.0415 PMC2124481

[32] Pfannes, K. R. & Baier, A. “Devil’s Gardens” in the Ecuadorian Amazon-Association of the allelopathic tree *Duroia hirsuta* (Rubiaceae) and its “gentle” ants. *Rev. Biol. Trop.* **50**, 293 (2002). 12298256

[33] Ribeiro, S. P., Espirito Santo, N. B., Delabie, J. H. C. & Majer, J. D. Competition, resources and the ant (Hymenoptera: Formicidae) mosaic: a comparison of upper and lower canopy. *Myrmecol. News* **18**, 113 (2013).

[34] Barriga. Community Structure and Ecological Specialization in Plant-Ant Interactions. Ph.D. Thesis, University of Arkansas, USA (2012).

[35] Barriga, P. A., Dormann, C. F., Gbur, E. E. & Sagers, C. L. Community structure and ecological specialization in plant–ant interactions. *J. Trop. Ecol.* **31**(4), 325 (2015). 10.1017/S0266467415000139

[36] Romoleroux, K., Foster, R., Valencia, R., Condit, R., Balslev, H. & Losos, E. Especies leñosas (dap ≥1 cm) encontradas en dos hectáreas de un bosque de la Amazonía ecuatoriana. *Estud. Divers. Ecol. Plantas* 189–215 (1997).

[37] Garwood, N. C. *et al.* Seasonality of reproduction in an ever-wet lowland tropical forest in Amazonian Ecuador. *Ecology* **104**(9), e4133 (2023). 37376710 10.1002/ecy.4133

[38] Valencia, R. *et al.* Tree species distributions and local habitat variation in the Amazon: large forest plot in eastern Ecuador. *J. Ecol.* **92**, 214 (2004). 10.1111/j.0022-0477.2004.00876.x

[39] Valencia, R., Condit, R.G., Foster, R.B., Romoleroux, K., VillaMuñoz, G., Svenning, J.C., *et al*. Yasuní Forest Dynamics Plot, Ecuador. In *Trop. Forest Divers. Dynamism* 609–628. University of Chicago Press (2004).

[40] Pérez, Á. J., Hernández, C., Romero-Saltos, H., & Valencia, R. Árboles emblemáticos de Yasuní, Ecuador. Escuela de Ciencias Biológicas, Pontificia Universidad Católica del Ecuador, 2014, ISBN 978-9942-20-260-4.

[41] Pinter-Wollman, N. Personality in social insects: How does worker personality determine colony personality?. *Curr. Zool.* **58**(4), 580–588 (2012). 10.1093/czoolo/58.4.580

[42] Bockoven, A. A., Wilder, S. M. & Eubanks, M. D. Intraspecific variation among social insect colonies: persistent regional and colony–level differences in fire ant foraging behavior. *PLoS ONE* **10**(7), e0133868 (2015). 26197456 10.1371/journal.pone.0133868 PMC4510567

[43] Horna-Lowell, E., Neumann, K. M., O’Fallon, S., Rubio, A., & Pinter-Wollman, N. Personality of ant colonies (Hymenoptera: Formicidae)–underlying mechanisms and ecological consequences. *Myrmecol. News* **31** (2021).

[44] Bizerril, M. X. & Vieira, E. M. Azteca ants as antiherbivore agents of *Tococa formicaria* (Melastomataceae) in Brazilian Cerrado. *Stud. Neotrop. Fauna Environ.* **37**(2), 145 (2002). 10.1076/snfe.37.2.145.8585

[45] Handley, R., Ekbom, B. & Agren, J. Variation in trichome density and resistance against a specialist insect herbivore in natural populations of *Arabidopsis thaliana*. *Ecol. Entomol.* **30**(3), 284 (2005). 10.1111/j.0307-6946.2005.00699.x

[46] Cornelissen, J. H. C. *et al.* A handbook of protocols for standardized and easy measurement of plant functional traits worldwide. *Aust. J. Bot.* **51**(4), 335 (2003). 10.1071/BT02124

[47] Pérez-Harguindeguy, N. *et al.* Corrigendum to: New handbook for standardized measurement of plant functional traits worldwide. *Aust. J. Bot.* **64**(8), 167 (2016). 10.1071/BT12225_CO

[48] Cobo-Quinche, J., Endara, M. J., Valencia, R., Muñoz-Upegui, D. & Cárdenas, R. E. Physical, but not chemical, antiherbivore defence expression is related to the clustered spatial distribution of tropical trees in an Amazonian forest. *Ecol. Evol.* **9**(4), 1750 (2019). 30847070 10.1002/ece3.4859 PMC6392389

[49] Wiggins, N. L., Forrister, D. L., Endara, M. J., Coley, P. D. & Kursar, T. A. Quantitative and qualitative shifts in defensive metabolites define chemical defence investment during leaf development in *Inga*, a genus of tropical trees. *Ecol. Evol.* **6**(2), 478 (2016). 26843932 10.1002/ece3.1896 PMC4729263

[50] Schuldt, A. *et al.* Plant traits affecting herbivory on tree recruits in highly diverse subtropical forests. *Ecol. Lett.* **15**(7), 732 (2012). 22548792 10.1111/j.1461-0248.2012.01792.x

[51] Getman-Pickering, Z. L. *et al*. LeafByte: A mobile application that measures leaf area and herbivory quickly and accurately. *Methods Ecol. Evol.*10.1111/2041-210X.13340 (2020).

[52] Cárdenas, R. E., Valencia, R., Kraft, N. J. B., Argoti, A. & Dangles, O. Plant traits predict inter- and intraspecific variation in susceptibility to herbivory in a hyperdiverse Neotropical rainforest tree community. *J. Ecol.* **102**(4), 939 (2014). 10.1111/1365-2745.12255

[53] Utreras, D. Defensas físicas y crecimiento secundario de *Duroia hirsuta* (Rubiaceae) en contraste al mutualismo que presenta con hormigas en el Parque Nacional Yasuní. [B.S. dissertation thesis]. Pontificia Universidad Católica del Ecuador, Quito, Ecuador (2022).

[54] Onoda, Y. *et al.* Global patterns of leaf mechanical properties. *Ecol. Lett.* **14**(3), 301 (2011). 21265976 10.1111/j.1461-0248.2010.01582.x

[55] Martins, D. *et al.* Triterpenes and the antimycobacterial activity of *Duroia macrophylla* Huber (Rubiaceae). *BioMed Res. Int.* **2013**, 605831 (2013). 23509750 10.1155/2013/605831 PMC3583080

[56] Page, J. E., Madrinan, S. & Towers, G. H. N. Identification of a plant growth inhibiting iridoid lactone from *Duroia hirsuta*, the allelopathic tree of the ‘Devil’s Garden’. *Experientia* **50**, 840–842 (1994). 10.1007/BF01956467

[57] Sotero, V., Suarez, P., Vela, J. E., de Sotero, D. G. & Fujii, Y. Allelochemicals of three Amazon plants identified by GC-MS. *Int. J. Eng. Appl. Sci.* **3**(2), 257731 (2016).

[58] Benton, H. P., Want, E. J. & Ebbels, T. M. D. Correction of mass calibration gaps in liquid chromatography-mass spectrometry metabolomics data. *Bioinformatics* **26**, 2488 (2010). 20671148 10.1093/bioinformatics/btq441

[59] Smith, C. A., Want, E. J., O’Maille, G., Abagyan, R. & Siuzdak, G. XCMS: Processing mass spectrometry data for metabolite profiling using nonlinear peak alignment, matching and identification. *Anal. Chem.* **78**, 779–787 (2006). 16448051 10.1021/ac051437y

[60] Tautenhahn, R., Boettcher, C. & Neumann, S. Highly sensitive feature detection for high resolution LC/MS. *BMC Bioinform.* **9**, 504 (2008). 10.1186/1471-2105-9-504 PMC2639432 19040729

[61] Brooks, M. E. *et al.* glmmTMB balances speed and flexibility among packages for zero-inflated generalized linear mixed modeling. *R J.* **9**(2), 378–400 (2017). 10.32614/RJ-2017-066

[62] Brooks, M. E., Bolker, B., Kristensen, K., Maechler, M., Magnusson, A, Skaug, H., Nielsen, A., Berg, C., and van Bentham, K. *glmmTMB: Generalized Linear Mixed Models Using Template Model Builder*. (2023) https://github.com/glmmTMB/glmmTMB.

[63] Bolker, B. M. *et al.* Generalized linear mixed models: a practical guide for ecology and evolution. *Trends Ecol. Evol.* **24**(3), 127–135 (2009). 19185386 10.1016/j.tree.2008.10.008

[64] Endara, M. J. *et al.* The role of plant secondary metabolites in shaping regional and local plant community assembly. *J. Ecol.* **110**(1), 34 (2021). 10.1111/1365-2745.13646

[65] R Core Team. *R: A language and environment for statistical computing.* R Foundation for Statistical Computing, Vienna, Austria (2022). https://www.R-project.org/.

[66] Suzuki R, Terada Y, Shimodaira H. *pvclust: Hierarchical Clustering with P-Values via Multiscale Bootstrap Resampling*. R package version 2.2-0. https://CRAN.R-project.org/package=pvclust.

[67] Hammer, Ø., Harper, D. A. T. & Ryan, P. D. PAST: Palaeontological Statistics software package for education and data analysis. *Palaeontol. Electron.* **4**(1), 1 (2001).

[68] Lumivero. *XLSTAT statistical and data analysis solution*. New York, USA. 2023. https://www.xlstat.com/es.

[69] Frederickson, M. E. & Gordon, D. M. The intertwined population biology of two Amazonian myrmecophytes and their symbiotic ants. *Ecology* **90**(6), 1595 (2009). 19569374 10.1890/08-0010.1

[70] Aljbory, Z. & Chen, M. S. Indirect plant defence against insect herbivores: a review. *Insect Sci.* **25**(1), 2 (2018). 28035791 10.1111/1744-7917.12436

[71] Kessler, A. & Heil, M. The multiple faces of indirect defences and their agents of natural selection. *Funct. Ecol.* **25**(2), 348 (2011). 10.1111/j.1365-2435.2010.01818.x

[72] Wright, S. J. Plant diversity in tropical forests: a review of mechanisms of species coexistence. *Oecologia* **130**(1), 1 (2002). 28547014 10.1007/s004420100809

[73] Pfannes, K. R. & Baier, A. “Devil’s Gardens” in the Ecuadorian Amazon-Association of the allelopathic tree *Duroia hirsuta* (Rubiaceae) and its “gentle” ants. *Rev. Biol. Trop.* **50**(1), 293–301 (2002). 12298256

[74] Bruna, E. M., Lapola, D. M. & Vasconcelos, H. L. Interspecific variation in the defensive responses of obligate plant-ants: Experimental tests and consequences for herbivory. *Oecologia* **138**(4), 558–565 (2004). 14689295 10.1007/s00442-003-1455-5

[75] Richards, L. A. & Coley, P. D. Seasonal and habitat differences affect the impact of food and predation on herbivores: a comparison between gaps and understory of a tropical forest. *Oikos* **116**(1), 31–40 (2007). 10.1111/j.2006.0030-1299.15043.x

[76] Choong, M. F. *et al.* Leaf fracture toughness and sclerophylly: their correlations and ecological implications. *New Phytol.* **121**(4), 597 (1992). 10.1111/j.1469-8137.1992.tb01131.x

[77] Dominy, N. J., Lucas, P. W. & Wright, S. J. Mechanics and chemistry of rainforest leaves: Canopy and understorey compared. *J. Exp. Bot.* **54**(390), 2007 (2003). 12867549 10.1093/jxb/erg224

[78] Westbrook, J. W. *et al.* What makes a leaf tough? Patterns of correlated evolution between leaf toughness traits and demographic rates among 197 shade-tolerant woody species in a neotropical forest. *Am. Nat.* **177**(6), 800 (2011). 21597256 10.1086/659963

[79] Alvarez-Clare, S. & Kitajima, K. Physical defence traits enhance seedling survival of neotropical tree species. *Funct. Ecol.* **21**(6), 1044 (2007). 10.1111/j.1365-2435.2007.01320.x

[80] Dominy, N. J. *et al.* In tropical lowland rainforests, monocots have tougher leaves than dicots and include a new kind of tough leaf. *Ann. Bot.* **101**(9), 1363 (2008). 18387969 10.1093/aob/mcn046 PMC2710255

[81] Kitajima, K. & Poorter, L. Tissue-level leaf toughness, but not lamina thickness, predicts sapling leaf lifespan and shade tolerance of tropical tree species. *New Phytol.* **186**(3), 708 (2010). 20298481 10.1111/j.1469-8137.2010.03212.x

[82] Coley, P. D. & Barone, J. A. Herbivory and plant defences in tropical forests. *Annu. Rev. Ecol. Syst.* **27**(1), 305 (1996). 10.1146/annurev.ecolsys.27.1.305

[83] Frederickson, M. E. *et al.* What happens when ants fail at plant defence?. *J. Ecol.* **101**, 400 (2013). 10.1111/1365-2745.12034

[84] Bixenmann, R. J., Coley, P. D., Weinhold, A. & Kursar, T. A. High herbivore pressure favors constitutive over induced defense. *Ecol. Evol.* **6**(17), 6037–6049 (2016). 27648224 10.1002/ece3.2208 PMC5016630

[85] Massad, T. J., Fincher, R. M., Smilanich, A. M. & Dyer, L. A quantitative evaluation of major plant defense hypotheses, nature versus nurture, and chemistry versus ants. *Arthropod-Plant Interact.* **5**, 125–139 (2011). 10.1007/s11829-011-9121-z

[86] Brenes-Arguedas, T. *et al.* Contrasting mechanisms of secondary metabolite accumulation during leaf development in two tropical tree species with different leaf expansion strategies. *Oecologia* **149**(1), 91–100 (2006). 16676208 10.1007/s00442-006-0423-2

[87] Kursar, T. A. & Coley, P. D. Convergence in defense syndromes of young leaves in tropical rainforests. *Biochem. Syst. Ecol.* **31**(8), 929–949 (2003). 10.1016/S0305-1978(03)00087-5

[88] Barton, K. E., Edwards, K. F. & Koricheva, J. Shifts in woody plant defense syndromes during leaf development. *Funct. Ecol.* **33**(11), 2095–2104 (2019). 10.1111/1365-2435.13435

[89] Montalvo-Yánez, S. G. Patrones de distribución de recursos en los árboles de los jardines del diablo: crecimiento y reproducción de la miremecofita dioica *Duroia hirsuta*. Tesis previa a la obtención del título de Licenciada en Ciencias Biológicas, Pontificia Universidad Católica del Ecuador, Quito, Ecuador (2022). http://repositorio.puce.edu.ec:80/handle/22000/20913.

[90] Sandoval-Molina, M. A., García-Franco, J. G., Díaz-Castelazo, C. & Janczur, M. K. Plant sex changes the outcome of ant–plant interactions in a facultative myrmecophytic cactus. *Funct. Ecol.* **37**(3), 778–790 (2023). 10.1111/1365-2435.14267

