# Supplementary material for: Long-term strict ant-plant mutualism identity characterises growth rate and leaf shearing resistance of an Amazonian myrmecophyte
Source: Sci Rep. 2024 Aug 1;14:17813. doi: 10.1038/s41598-024-67140-4 (PMC11294366; doi:10.1038/s41598-024-67140-4)

**Supplementary material**

**Fig. S1. Two trees of *D. hirsuta* in different microhabitats as a consequence of different ant associations.**


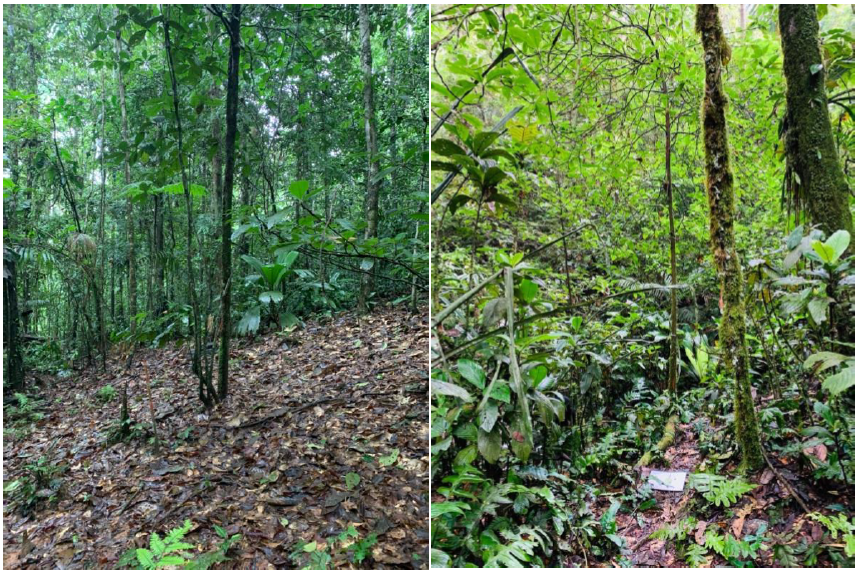


Left: *D. hirsuta* tree in a “Devil's Garden” in mutualism with *M. schumanni*.

Right: *D. hirsuta* tree in the understory in mutualism with *Azteca* spp.

Images taken from [53].

**References**

53. Utreras-Echeverría, D.S. Defensas físicas y crecimiento secundario de *Duroia hirsuta* (Rubiaceae) en contraste al mutualismo que presenta con hormigas en el Parque Nacional Yasuní. Tesis previa a la obtención del título de Licenciada en Ciencias Biológicas, Pontificia Universidad Católica del Ecuador, Quito, Ecuador (2022). <https://repositorio.puce.edu.ec/handle/123456789/11>

**Table S1.** Ultraperformance liquid chromatographic separation of peaks using a 22.5-min reverse-phase gradient with water and acetonitrile as the mobile and stationary phases, respectively (modified from [49])

| Time (minutes) | Flow Rate (ml/min) | % Acetonitrile (+0.1% formic acid) |
| --- | --- | --- |
| 1 | 0.5 | 2 |
| 2 | 0.5 | 4 |
| 6 | 0.5 | 16 |
| 10 | 0.5 | 40 |
| 14 | 0.5 | 70 |
| 16 | 0.5 | 98 |
| 17 | 0.5 | 98 |
| 19 | 0.5 | 2 |
| 22.5 | 0.5 | 2 |

**References**

49. Wiggins, N. L., Forrister, D. L., Endara, M. J., Coley, P. D., & Kursar, T. A. Quantitative and qualitative shifts in defensive metabolites define chemical defence investment during leaf development in *Inga*, a genus of tropical trees. *Ecol. Evol.*, 6(2), 478 (2016).

**Table S2.** Spearman’s rho significant correlations at different radii on conspecific neighbour counts used to observe the effects of *D. hirsuta*'s neighbouring trees on their growth rate.

|  | | | | | | |
| --- | --- | --- | --- | --- | --- | --- |
|  | | | **5 m** | **10 m** | **15 m** | **20 m** |
|  | **5 m** | Correlation Coefficient |  | 0.853^**^ | 0.797^**^ | 0.740^**^ |
|  |  | Sig. (2-tailed) |  | *p* < 0.01 | *p* < 0.01 | *p* < 0.01 |
|  |  | N |  | 58 | 58 | 58 |
|  | **10 m** | Correlation Coefficient | 0.853^**^ |  | 0.955^**^ | 0.897^**^ |
|  |  | Sig. (2-tailed) | *p* < 0.01 |  | *p* < 0.01 | *p* < 0.01 |
|  |  | N | 58 |  | 58 | 58 |
|  | **15 m** | Correlation Coefficient | 0.797^**^ | 0.955^**^ |  | 0.956^**^ |
|  |  | Sig. (2-tailed) | *p* < 0.01 | *p* < 0.01 |  | *p* < 0.01 |
|  |  | N | 58 | 58 |  | 58 |
|  | **20 m** | Correlation Coefficient | 0.740^**^ | 0.897^**^ | 0.956^**^ |  |
|  |  | Sig. (2-tailed) | *p* < 0.01 | *p* < 0.01 | *p* < 0.01 |  |
|  |  | N | 58 | 58 | 58 |  |
|  | | | | | | |

**Table S3.** Raw data used for GLMM-TMB models. Herbivory, LDMC and Shearing resistance are averages of multiple measurements for each tree. Original Herbivory percentage values here were converted to 0–1 scale proportions (N = 870) and applied arcsine-square root transformation; missing values (n = 6) were replaced with subsamples averages.
RGR was calculated using the DBH of each target tree at *t*_1_ (= 2019) relative to the DBH at *t*_0_ (= 2007). Neighbourhood effects on predicted variables used conspecific plant neighbours within 10-m radius.
RGR: relative growth rate; LDMC: leaf dry matter content; Longitude, Latitude: Cartesian coordinates representing the plant's location within the plot (i.e., growing site).

| **Tree tag** | **Ant identity** | **RGR** | **Herbivory** | **LDMC** | **Shearing resistance** | **Longitude** | **Latitude** | **Habitat** | **Neighbours 1m** | **Neighbours 5m** | **Neighbours 10m** | **Neighbours 15m** | **Neighbours 20m** |
| --- | --- | --- | --- | --- | --- | --- | --- | --- | --- | --- | --- | --- | --- |
| 1828 | Azteca | 0.018915187 | 2.953223368 | 3.285492893 | 0.000784234 | 1.55 | 133.37 | ridge | 1 | 1 | 1 | 1 | 1 |
| 14829 | Myrmelachista | 0.006319162 | 3.891879867 | 3.080005163 | 0.000745228 | 25.51 | 405.13 | ridge | 1 | 1 | 1 | 1 | 1 |
| 20542 | Myrmelachista | 0.020651701 | 5.845448944 | 3.467011028 | 0.001722084 | 53.71 | 31.28 | ridge | 1 | 2 | 2 | 2 | 2 |
| 20545 | Myrmelachista | 0.005648148 | 4.573225179 | 4.228957986 | 0.001442345 | 51.76 | 31.83 | ridge | 1 | 2 | 2 | 2 | 2 |
| 33609 | Azteca | 0.011167428 | 3.229346871 | 3.385239752 | 0.000907726 | 62.7 | 268.03 | valley | 1 | 1 | 1 | 1 | 1 |
| 33958 | Myrmelachista | 0.02972992 | 4.519815855 | 2.977887487 | 0.000893946 | 76.33 | 294.96 | ridge | 2 | 8 | 8 | 8 | 8 |
| 35759 | Myrmelachista | 0.052959246 | 5.231256429 | 3.42243966 | 0.001659159 | 71.952 | 441.44 | ridge | 1 | 2 | 6 | 7 | 7 |
| 41129 | Myrmelachista | 0.022629415 | 0.742066891 | 3.369852181 | 0.001107158 | 85.09 | 89.33 | ridge | 1 | 1 | 1 | 1 | 2 |
| 41818 | Azteca | 0.00004270 | 2.786993647 | 2.936989539 | 0.000747703 | 93.44 | 121.42 | ridge | 1 | 1 | 1 | 1 | 1 |
| 42418 | Myrmelachista | 0.073556071 | 1.339353862 | 2.999773319 | 0.000699473 | 94.48 | 161.35 | ridge | 1 | 1 | 1 | 1 | 1 |
| 54633 | Myrmelachista | 0.124511947 | 5.573074152 | 3.526955818 | 0.001307352 | 105.73 | 352.68 | ridge | 1 | 4 | 4 | 4 | 4 |
| 56316 | Azteca | 0.004563305 | 4.777999773 | 3.323679007 | 0.000722993 | 119.44 | 498.64 | ridge | 1 | 3 | 3 | 3 | 3 |
| 56317 | Azteca | 0.006382813 | 4.39972082 | 3.260636169 | 0.000871078 | 119.98 | 497.27 | ridge | 1 | 3 | 3 | 3 | 3 |
| 60345 | Azteca | 0.025254737 | 3.252481966 | 2.956856737 | 0.000662527 | 121.11 | 38.22 | ridge | 1 | 1 | 2 | 3 | 4 |
| 63466 | Azteca | 0.00000000 | 3.430615143 | 3.426070451 | 0.001169831 | 120.35 | 307.32 | ridge | 1 | 3 | 5 | 9 | 9 |
| 63472 | Azteca | 0.009605334 | 2.835599674 | 3.848315448 | 0.00190429 | 123.47 | 307.75 | ridge | 1 | 4 | 8 | 8 | 9 |
| 63488 | Myrmelachista | 0.000537587 | 5.158028034 | 2.319283111 | 0.001532172 | 131.28 | 302.15 | ridge | 2 | 2 | 4 | 7 | 8 |
| 63605 | Azteca | 0.001783637 | 2.688005189 | 3.762161207 | 0.001711888 | 125.16 | 315.46 | ridge | 1 | 2 | 6 | 8 | 8 |
| 63633 | Azteca | 0.01267037 | 5.234217119 | 3.731480428 | 0.001771078 | 124.84 | 317.32 | ridge | 1 | 2 | 4 | 6 | 8 |
| 70481 | Myrmelachista | 0.021136733 | 3.810106861 | 3.16448579 | 0.000957645 | 150.58 | 42.47 | ridge | 1 | 3 | 3 | 3 | 3 |
| 72096 | Myrmelachista | 0.004328293 | 2.344503842 | 3.4667681 | 0.001292352 | 145.58 | 179.16 | ridge | 1 | 4 | 8 | 9 | 9 |
| 72146 | Azteca | 0.003761759 | 2.268262503 | 2.944931898 | 0.001169135 | 142.39 | 184.42 | valley | 1 | 4 | 9 | 9 | 9 |
| 72264 | Azteca | 0.000538502 | 3.799671201 | 3.193210869 | 0.001088751 | 146.83 | 190.25 | valley | 1 | 2 | 7 | 9 | 9 |
| 75177 | Myrmelachista | 0.020687808 | 2.470368668 | 3.501854485 | 0.000797895 | 151.56 | 424.93 | ridge | 1 | 3 | 6 | 6 | 6 |
| 80439 | Myrmelachista | 0.000252717 | 1.396713957 | 3.7374617 | 0.001249092 | 163.86 | 126.44 | ridge | 1 | 1 | 1 | 1 | 1 |
| 83724 | Azteca | 0.00000000 | 6.788042356 | 3.478299758 | 0.002775185 | 160.4 | 324.42 | ridge | 1 | 1 | 1 | 2 | 4 |
| 86016 | Myrmelachista | 0.002542918 | 6.287913588 | 2.896413356 | 0.001587437 | 169.64 | 486.61 | ridge | 1 | 2 | 6 | 7 | 9 |
| 86069 | Myrmelachista | 0.031392593 | 2.637475702 | 3.521072428 | 0.003079479 | 177.91 | 489 | ridge | 2 | 3 | 6 | 8 | 12 |
| 93405 | Azteca | 0.00000000 | 6.007884787 | 3.045641391 | 0.001152698 | 191.46 | 286.28 | ridge | 1 | 2 | 5 | 7 | 7 |
| 96147 | Myrmelachista | 0.064360138 | 3.551572185 | 3.048247801 | 0.002295118 | 185.56 | 471.21 | ridge | 1 | 4 | 6 | 7 | 11 |
| 96200 | Myrmelachista | 0.026224899 | 5.499858323 | 3.099175256 | 0.001566466 | 181.74 | 471.57 | ridge | 1 | 4 | 6 | 9 | 12 |
| 104981 | Myrmelachista | 0.006533531 | 2.682935532 | 3.351142823 | 0.003514519 | 208.73 | 424.88 | ridge | 1 | 2 | 4 | 4 | 4 |
| 105004 | Myrmelachista | 0.021667188 | 1.548595896 | 3.597264276 | 0.002541436 | 210.58 | 424.03 | ridge | 1 | 2 | 4 | 4 | 4 |
| 106016 | Myrmelachista | 0.11584300 | 4.143986244 | 3.525829544 | 0.005196323 | 201.33 | 490.37 | ridge | 1 | 1 | 2 | 2 | 2 |
| 114493 | Azteca | 0.014095372 | 4.184461306 | 3.025033826 | 0.001047884 | 226.74 | 386.89 | ridge | 1 | 3 | 3 | 3 | 3 |
| 116849 | Azteca | 0.083125926 | 2.718806864 | 3.095965169 | 0.001036389 | 224.92 | 387.82 | ridge | 1 | 3 | 3 | 3 | 3 |
| 120005 | Myrmelachista | 0.017585289 | 3.038013699 | 3.587034271 | 0.000887796 | 240.45 | 2.54 | ridge | 1 | 1 | 1 | 1 | 1 |
| 122367 | Azteca | 0.010882337 | 6.063799836 | 2.430807432 | 0.001063185 | 241 | 192.15 | valley | 1 | 1 | 1 | 1 | 1 |
| 130034 | Azteca | 0.00000000 | 1.779279018 | 2.411420194 | 0.000570712 | 264.62 | 8.51 | ridge | 1 | 1 | 1 | 1 | 1 |
| 131103 | Azteca | 0.002150449 | 3.126917346 | 2.790459994 | 0.001581782 | 260.39 | 73.33 | ridge | 1 | 1 | 1 | 1 | 1 |
| 142786 | Myrmelachista | 0.06647872 | 3.290295529 | 3.396797679 | 0.0010551 | 290.93 | 236.34 | valley | 1 | 1 | 1 | 1 | 2 |
| 151054 | Myrmelachista | 0.04687500 | 4.601375146 | 3.317264228 | 0.001238999 | 305.67 | 80.78 | ridge | 1 | 1 | 1 | 1 | 1 |
| 152726 | Azteca | 0.018521091 | 3.150661497 | 3.281169082 | 0.001192382 | 303.93 | 219.32 | valley | 1 | 1 | 1 | 3 | 4 |
| 163682 | Azteca | 0.03270000 | 2.156537981 | 2.558584617 | 0.000543644 | 338.24 | 299.76 | ridge | 1 | 1 | 1 | 1 | 1 |
| 165617 | Myrmelachista | 0.056169822 | 6.771947466 | 3.961015739 | 0.002444763 | 338.54 | 440.83 | ridge | 2 | 2 | 6 | 9 | 10 |
| 175040 | Myrmelachista | 0.077846651 | 2.759278748 | 2.049269998 | 0.001025644 | 346.1 | 378.81 | ridge | 2 | 2 | 3 | 3 | 6 |
| 175337 | Azteca | 0.011167428 | 4.532009748 | 3.267068796 | 0.000981026 | 343.03 | 396.47 | ridge | 1 | 1 | 1 | 1 | 3 |
| 183513 | Myrmelachista | 0.045817833 | 3.43013567 | 4.239722644 | 0.001104598 | 370.44 | 289.1 | ridge | 1 | 2 | 2 | 2 | 2 |
| 203225 | Myrmelachista | 0.023644313 | 5.569547675 | 3.577846566 | 0.001306585 | 406.9 | 265.1 | ridge | 1 | 1 | 1 | 1 | 1 |
| 204022 | Azteca | 0.058642659 | 3.354437008 | 2.99825468 | 0.00081835 | 407.86 | 336.08 | ridge | 2 | 2 | 3 | 4 | 4 |
| 204023 | Azteca | 0.032419218 | 3.624624124 | 2.769633556 | 0.000797989 | 407.3 | 336.52 | ridge | 2 | 2 | 2 | 4 | 4 |
| 212229 | Azteca | 0.021396584 | 3.331217171 | 2.174094765 | 0.000701591 | 430.46 | 170.53 | valley | 1 | 1 | 2 | 2 | 2 |
| 214845 | Azteca | 0.010433886 | 5.51952158 | 2.949381398 | 0.002051742 | 433.74 | 419.97 | ridge | 1 | 1 | 2 | 2 | 3 |
| 223490 | Myrmelachista | 0.046262976 | 4.183301755 | 3.078617936 | 0.00114713 | 442.15 | 253.76 | valley | 1 | 4 | 4 | 4 | 7 |
| 224471 | Azteca | 0.059238667 | 3.975375295 | 7.907142153 | 0.000566778 | 446.77 | 351.21 | ridge | 1 | 4 | 5 | 5 | 5 |
| 237805 | Myrmelachista | 0.034391837 | 2.199883754 | 3.281220435 | 0.000784555 | 460.476 | 150.6 | valley | 1 | 4 | 6 | 6 | 6 |
| 240260 | Azteca | 0.00680000 | 1.543588089 | 3.582859667 | 0.000864185 | 489.54 | 15.44 | ridge | 1 | 1 | 2 | 2 | 2 |
| 240409 | Azteca | 0.066065005 | 2.803876216 | 3.701078326 | 0.001071474 | 488.38 | 21.33 | ridge | 1 | 1 | 2 | 2 | 2 |

**Table S4.** Individual trees of *Duroia hirsuta* status of (A) trees found in exclusive mutualism with *Myrmelachista schumanni* and *Azteca* spp. since censused as recruits (i.e., when DAP reaches 1 cm, the moment when they form domatia in their branches to host ants), and (B) individual trees found with no mutualism with ants or with short term mutualism mainly with *M. schumanni* and *Azteca* spp.

NA means ‘No Ant’ ever recorded. *presence in tree: 2010–2013.

**(A) (B)**

| **Tag** | **Status** | **Ant species 2007 - 2019 (if noted, last year present in tree)** |  | **Tag** | **Status** | **Temporal ant species 2007-2019 (1 up to 4 years of ants’ presence)** |
| --- | --- | --- | --- | --- | --- | --- |
| 367 | Alive | *Azteca* |  | 3239 | Dead | *Azteca* |
| 1828 | Alive | *Azteca* |  | 6668 | Dead | *Myrmelachista* |
| 7718 | Alive | *Myrmelachista* |  | 14045 | Dead | NA |
| 9959 | Alive | *Myrmelachista* in 2016 |  | 16363 | Dead | NA |
| 14198 | Alive | *Myrmelachista* |  | 35749 | Dead | NA |
| 14829 | Alive | *Myrmelachista* |  | 35771 | Dead | *Myrmelachista* |
| 17698 | Alive | *Myrmelachista* |  | 35813* | Alive | *Myrmelachista* |
| 20542 | Alive | *Myrmelachista* |  | 38732 | Dead | NA |
| 20545 | Alive | *Myrmelachista* |  | 40945 | Dead | NA |
| 33609 | Alive | *Azteca* |  | 50551 | Dead | *Brachymyrmex*/*Azteca* |
| 33951 | Alive | *Myrmelachista* |  | 53826 | Dead | NA |
| 33952 | Alive | *Myrmelachista* |  | 62863 | Dead | NA |
| 33953 | Alive | *Myrmelachista* |  | 65973 | Dead | NA |
| 33954 | Alive | *Myrmelachista* |  | 63636 | Dead | *Azteca*/*Pseudomyrmex* |
| 33958 | Alive | *Myrmelachista* |  | 69968 | Dead | NA |
| 33959 | Alive | *Myrmelachista* |  | 72149 | Dead | NA |
| 35687 | Alive | *Myrmelachista* |  | 82515 | Dead | NA |
| 35759 | Alive | *Myrmelachista* |  | 83223 | Dead | NA |
| 36285 | Alive | *Myrmelachista* |  | 83645 | Dead | NA |
| 37241 | Alive | *Myrmelachista* |  | 93425 | Dead | NA |
| 37322 | Alive | *Myrmelachista* |  | 93442 | Dead | NA |
| 37768 | Alive | *Myrmelachista* |  | 93585 | Dead | *Crematogaster*/*Myrmelachista* |
| 41129 | Alive | *Myrmelachista* |  | 102003 | Dead | NA |
| 41818 | Alive | *Azteca* |  | 104947 | Dead | NA |
| 42418 | Alive | *Myrmelachista* |  | 123610 | Dead | *Azteca* |
| 44586 | Alive | *Myrmelachista* |  | 140305 | Dead | NA |
| 50367 | Alive | *Azteca* |  | 152941 | Dead | *Myrmelachista* |
| 54633 | Alive | *Myrmelachista* |  | 154311 | Dead | NA |
| 54634 | Alive | *Myrmelachista* |  | 155967 | Dead | NA |
| 54661 | Alive | *Myrmelachista* |  | 164022 | Dead | *Myrmelachista* |
| 54672 | Alive | *Myrmelachista* |  | 165310 | Dead | NA |
| 56316 | Alive | *Azteca* |  | 165483 | Dead | NA |
| 56317 | Alive | *Azteca* |  | 165484 | Dead | *Azteca*/*Pheidole* |
| 60345 | Alive | *Azteca* |  | 165509 | Dead | NA |
| 63466 | Alive | *Azteca* |  | 171084 | Dead | NA |
| 63472 | Alive | *Azteca* |  | 177194 | Dead | *Myrmelachista* |
| 63473 | Alive | *Azteca* |  | 190074 | Dead | NA |
| 63488 | Alive | *Myrmelachista* |  | 195534 | Dead | NA |
| 63489 | Alive | *Myrmelachista* |  | 222710 | Dead | *Azteca* |
| 63605 | Alive | *Azteca* |  | 224315 | Dead | *Myrmelachista* |
| 63633 | Alive | *Azteca* |  | 225323 | Dead | NA |
| 64614 | Alive | *Myrmelachista* |  | 227517 | Dead | NA |
| 68367 | Alive | *Myrmelachista* |  | 231162 | Dead | *Azteca* |
| 70481 | Alive | *Myrmelachista* |  | 235441 | Dead | *Myrmelachista* |
| 72096 | Alive | *Myrmelachista* |  |  |  |  |
| 72146 | Alive | *Azteca* in 2016 |  |  |  |  |
| 72168 | Alive | *Azteca* |  |  |  |  |
| 72264 | Alive | *Azteca* |  |  |  |  |
| 73997 | Alive | *Azteca* |  |  |  |  |
| 74229 | Alive | *Azteca* |  |  |  |  |
| 74552 | Alive | *Myrmelachista* |  |  |  |  |
| 75157 | Alive | *Myrmelachista* |  |  |  |  |
| 75177 | Alive | *Myrmelachista* |  |  |  |  |
| 76146 | Alive | *Myrmelachista* |  |  |  |  |
| 76407 | Alive | *Azteca* |  |  |  |  |
| 76874 | Alive | *Myrmelachista* |  |  |  |  |
| 76875 | Alive | *Myrmelachista* |  |  |  |  |
| 77062 | Alive | *Myrmelachista* |  |  |  |  |
| 80207 | Alive | *Myrmelachista* |  |  |  |  |
| 80439 | Alive | *Myrmelachista* |  |  |  |  |
| 81367 | Alive | *Myrmelachista* |  |  |  |  |
| 83148 | Alive | *Myrmelachista* |  |  |  |  |
| 83724 | Alive | *Azteca* |  |  |  |  |
| 84978 | Alive | *Myrmelachista* |  |  |  |  |
| 86012 | Alive | *Myrmelachista* |  |  |  |  |
| 86016 | Alive | *Myrmelachista* |  |  |  |  |
| 86064 | Alive | *Myrmelachista* |  |  |  |  |
| 86069 | Alive | *Myrmelachista* |  |  |  |  |
| 86070 | Alive | *Myrmelachista* |  |  |  |  |
| 86081 | Alive | *Myrmelachista* |  |  |  |  |
| 86087 | Alive | *Myrmelachista* |  |  |  |  |
| 86702 | Alive | *Myrmelachista* |  |  |  |  |
| 90376 | Dead | *Myrmelachista* in 2016 |  |  |  |  |
| 93405 | Alive | *Azteca* |  |  |  |  |
| 93517 | Alive | *Myrmelachista* |  |  |  |  |
| 96147 | Alive | *Myrmelachista* |  |  |  |  |
| 96151 | Alive | *Myrmelachista* |  |  |  |  |
| 96180 | Alive | *Myrmelachista* |  |  |  |  |
| 96200 | Alive | *Myrmelachista* |  |  |  |  |
| 98316 | Alive | *Myrmelachista* |  |  |  |  |
| 101953 | Alive | *Myrmelachista* |  |  |  |  |
| 104853 | Alive | *Myrmelachista* |  |  |  |  |
| 104981 | Alive | *Myrmelachista* |  |  |  |  |
| 105004 | Alive | *Myrmelachista* |  |  |  |  |
| 105823 | Alive | *Myrmelachista* |  |  |  |  |
| 106016 | Alive | *Myrmelachista* |  |  |  |  |
| 112888 | Alive | *Myrmelachista* |  |  |  |  |
| 113626 | Alive | *Myrmelachista* |  |  |  |  |
| 114493 | Alive | *Azteca* |  |  |  |  |
| 116668 | Alive | *Myrmelachista* |  |  |  |  |
| 116849 | Alive | *Azteca* |  |  |  |  |
| 116850 | Alive | *Azteca* |  |  |  |  |
| 117070 | Alive | *Myrmelachista* |  |  |  |  |
| 120005 | Alive | *Myrmelachista* |  |  |  |  |
| 122367 | Alive | *Azteca* |  |  |  |  |
| 130034 | Alive | *Azteca* |  |  |  |  |
| 130516 | Alive | *Myrmelachista* (Azteca in 2017) |  |  |  |  |
| 131103 | Alive | *Azteca* |  |  |  |  |
| 133516 | Alive | *Azteca* |  |  |  |  |
| 135458 | Alive | *Azteca* |  |  |  |  |
| 142786 | Alive | *Myrmelachista* |  |  |  |  |
| 143781 | Alive | *Myrmelachista* |  |  |  |  |
| 143804 | Alive | *Myrmelachista* |  |  |  |  |
| 145794 | Alive | *Myrmelachista* |  |  |  |  |
| 147829 | Alive | *Myrmelachista* |  |  |  |  |
| 151054 | Alive | *Myrmelachista* |  |  |  |  |
| 152726 | Alive | *Azteca* |  |  |  |  |
| 152942 | Alive | *Azteca* |  |  |  |  |
| 152944 | Alive | *Myrmelachista* in 2018 |  |  |  |  |
| 158125 | Alive | *Myrmelachista* |  |  |  |  |
| 160505 | Alive | *Azteca* |  |  |  |  |
| 161123 | Alive | *Azteca* in 2014 |  |  |  |  |
| 162031 | Alive | *Myrmelachista* |  |  |  |  |
| 162055 | Alive | *Azteca in 2016* |  |  |  |  |
| 163682 | Alive | *Azteca* |  |  |  |  |
| 164102 | Alive | *Myrmelachista* |  |  |  |  |
| 164457 | Alive | *Myrmelachista* |  |  |  |  |
| 164710 | Alive | *Myrmelachista* |  |  |  |  |
| 165573 | Alive | *Myrmelachista* |  |  |  |  |
| 165617 | Alive | *Myrmelachista* |  |  |  |  |
| 168064 | Alive | *Myrmelachista* |  |  |  |  |
| 171532 | Alive | *Myrmelachista* |  |  |  |  |
| 171534 | Alive | *Myrmelachista* |  |  |  |  |
| 171569 | Alive | *Myrmelachista* |  |  |  |  |
| 173281 | Alive | *Azteca* |  |  |  |  |
| 175040 | Alive | *Myrmelachista* |  |  |  |  |
| 175042 | Alive | *Myrmelachista* |  |  |  |  |
| 175337 | Alive | *Azteca* |  |  |  |  |
| 177387 | Alive | *Myrmelachista* |  |  |  |  |
| 177456 | Alive | *Myrmelachista* |  |  |  |  |
| 181130 | Alive | *Myrmelachista* |  |  |  |  |
| 181600 | Alive | *Myrmelachista* |  |  |  |  |
| 183513 | Alive | *Myrmelachista* |  |  |  |  |
| 183995 | Alive | *Azteca* |  |  |  |  |
| 186874 | Alive | *Myrmelachista* |  |  |  |  |
| 192114 | Alive | *Azteca* |  |  |  |  |
| 193020 | Alive | *Azteca* |  |  |  |  |
| 194034 | Alive | *Myrmelachista* |  |  |  |  |
| 195711 | Alive | *Azteca* |  |  |  |  |
| 196307 | Alive | *Myrmelachista* |  |  |  |  |
| 200158 | Alive | *Myrmelachista* |  |  |  |  |
| 200706 | Alive | *Azteca* |  |  |  |  |
| 201849 | Alive | *Myrmelachista* |  |  |  |  |
| 203225 | Alive | *Myrmelachista* |  |  |  |  |
| 203875 | Alive | *Myrmelachista* in 2016 |  |  |  |  |
| 204022 | Alive | *Azteca* |  |  |  |  |
| 204023 | Alive | *Azteca* |  |  |  |  |
| 210259 | Alive | *Myrmelachista* |  |  |  |  |
| 212616 | Alive | *Myrmelachista* |  |  |  |  |
| 213868 | Alive | *Myrmelachista* |  |  |  |  |
| 216521 | Alive | *Myrmelachista* |  |  |  |  |
| 223386 | Alive | *Myrmelachista* |  |  |  |  |
| 223459 | Alive | *Myrmelachista* |  |  |  |  |
| 223480 | Alive | *Myrmelachista* |  |  |  |  |
| 223486 | Alive | *Myrmelachista* |  |  |  |  |
| 223490 | Alive | *Myrmelachista* |  |  |  |  |
| 223677 | Alive | *Myrmelachista* |  |  |  |  |
| 224316 | Alive | *Azteca* |  |  |  |  |
| 224471 | Alive | *Azteca* in 2016 |  |  |  |  |
| 230039 | Alive | *Myrmelachista* |  |  |  |  |
| 231293 | Dead | *Azteca* in 2015 |  |  |  |  |
| 232269 | Alive | *Myrmelachista* |  |  |  |  |
| 240260 | Alive | *Azteca* |  |  |  |  |
| 240409 | Alive | *Azteca* |  |  |  |  |

**Table S5.** Individual trees of *Duroia hirsuta* showing the shifts in ant mutualism over the years since first censused as recruits (i.e., when DAP reaches 1 cm, the moment when they form domatia in their branches to host ants). By 2019, one out of 52 followed individuals had died since it was first censused. NA means ‘No Ant’ recorded in that specific range of time.
*the only individual that survived after ≥5 years with total absence of ants. This tree was found in dire condition, showing damaged or broken branches and a scarce number of healthy leaves.

| **Tag** | **Status** | **Ants species during the years** |
| --- | --- | --- |
| 6752 | Alive | *Solenopsis* (2015-2016); NA (2017-2019) |
| 10844* | Alive | *Solenopsis* (2013-2014); NA (2015-2019) |
| 11024 | Alive | *Azteca* (2010-2013); *Brachymyrmex* (2014); *Crematogaster* (2015); *Azteca* (2016); NA (2017-2019) |
| 13662 | Alive | *Pseudomyrmex* (2015); NA (2016-2019) |
| 13663 | Alive | *Crematogaster* (2015); NA (2016-2019) |
| 26175 | Alive | *Pseudomyrmex* (2013); *Pachycondyla* (2014); *Azteca* (2015-2016); NA (2017-2019) |
| 35754 | Alive | *Pheidole* (2013-2015); NA (2016-2019) |
| 45830 | Alive | *Solenopsis* (2016); *Crematogaster* (2017-2019) |
| 50320 | Alive | *Brachymyrmex* (2014); *Pheidole* (2015); *Azteca* (2018-2019) |
| 50551 | Alive | *Brachymyrmex* (2014); NA (2015-2019) |
| 54634 | Alive | *Myrmelachista* (2010-2012); *Crematogaster* (2013-2015); *Myrmelachista* (2016-2019) |
| 54672 | Alive | *Crematogaster* (2013); NA (2014-2019) |
| 74229 | Alive | *Crematogaster* (2013-2014); *Azteca* (2018-2019) |
| 83130 | Alive | *Brachymyrmex* (2014-2019) |
| 83837 | Alive | *Myrmelachista* (2010-2013); *Pheidole* (2014); NA (2015-2019) |
| 92488 | Alive | *Pheidole* (2013); NA (2014-2019) |
| 92541 | Alive | *Azteca* (2010-2016); *Wasmannia* (2017-2019) |
| 93434 | Alive | *Azteca* (2010-2015); *Crematogaster* (2016-2019) |
| 114051 | Alive | *Azteca* (2013-2016); *Crematogaster*/*Brachymyrmex* (2017); *Myrmelachista* (2018-2019) |
| 141412 | Alive | *Azteca* (2010-2012); *Solenopsis* (2013-2015); *Crematogaster* (2016-2018); NA (2019) |
| 152942 | Alive | *Azteca* (2010-2015); *Crematogaster* (2016-2019) |
| 152943 | Alive | *Myrmelachista* (2010-2016); *Pheidole* (2017-2018); *Solenopsis* (2019) |
| 155805 | Alive | *Azteca* (2010-2013); *Wasmannia*/*Brachymyrmex* (2014-2015); *Azteca*/*Wasmannia*/*Pseudomyrmex* (2016); *Wasmannia* (2017-2019) |
| 155871 | Dead | *Azteca* (2010-2014); *Solenopsis* (2015); *Azteca* (2018-2019) |
| 155959 | Alive | Monomorium (2014); *Wasmannia* (2015-2016); *Solenopsis* (2017-2019) |
| 155981 | Alive | *Wasmannia* (2014-2017); *Solenopsis* (2018-2019) |
| 156975 | Alive | *Crematogaster* (2016); *Pachycondyla* (2017-2019) |
| 165484 | Alive | *Azteca* (2010-2013); *Pheidole* (2014-2018); NA (2019) |
| 165507 | Alive | *Solenopsis* (2013); *Wasmannia*/*Monomorium* (2014); *Solenopsis* (2015-2019) |
| 165553 | Alive | *Azteca* (2010-2012); *Wasmannia* (2013-2015); *Myrmelachista* (2016-2019) |
| 166141 | Alive | *Myrmelachista* (2010-2013); *Crematogaster* (2014-2016 (+*Monomorium* 2016)); *Azteca* (2017-2019) |
| 166489 | Alive | *Myrmelachista* (2013-2015); *Wasmannia* (2016); NA (2017-2019) |
| 172906 | Alive | *Myrmelachista* (2013); *Solenopsis* (2014); *Myrmelachista* (2015-2016); *Brachymyrmex* (2017-2019) |
| 187136 | Alive | *Solenopsis* (2016-2017); *Azteca* (2018-2019) |
| 195334 | Alive | *Crematogaster*/*Brachymyrmex* (2013); *Crematogaster* (2014-2017); *Azteca* (2018-2019) |
| 195646 | Alive | *Dolichoderus*/*Brachymyrmex* (2013); *Pseudomyrmex* (2014); *Camponotus* (2015); *Crematogaster* (2017) |
| 200706 | Alive | *Myrmelachista* (2007-2013); *Solenopsis* (2014-2016 (+*Brachymyrmex* 2016)); *Azteca* (2017-2019) |
| 200707 | Alive | *Myrmelachista* (2010-2013); *Solenopsis* (2014-2016); *Wasmannia* (2017-2019) |
| 202786 | Alive | *Solenopsis* (2017-2019) |
| 203875 | Alive | *Pseudomyrmex* (2013-2015); *Myrmelachista* (2016); NA (2017-2019) |
| 205099 | Alive | *Azteca*/*Brachymyrmex* (2013); *Brachymyrmex*/*Nylanderia* (2014); *Brachymyrmex*/*Crematogaster* (2016); *Brachymyrmex* (2017-2019) |
| 212229 | Alive | *Crematogaster* (2014); *Azteca* (2015-2017); NA (2018-2019) |
| 214719 | Alive | *Crematogaster* (2010); *Brachymyrmex* (2013-2014); *Crematogaster* (2015); *Azteca* (2016); *Myrmelachista* (2017-2019) |
| 214845 | Alive | *Brachymyrmex*/*Crematogaster* (2014); *Brachymyrmex* (2015); *Azteca* (2016-2019) |
| 215006 | Alive | *Myrmelachista* (2013); *Pachycondyla* (2014); *Crematogaster*/*Cephalotes* (2016); *Myrmelachista* (2017-2019) |
| 215566 | Alive | *Crematogaster*/*Pachycondyla* (2014); *Azteca* (2015-2016); *Crematogaster* (2017-2019) |
| 215567 | Alive | *Pseudomyrmex* (2014); NA (2017-2019) |
| 215834 | Alive | *Crematogaster* (2013); *Crematogaster*/*Cephalotes* (2014); *Solenopsis*/*Crematogaster*/Pachycondyla (2015); *Brachymyrmex* (2016-2019) |
| 224313 | Alive | *Myrmelachista* (2010-2013); *Camponotus* (2014-2016); *Azteca* (2017-2019) |
| 224521 | Alive | *Myrmelachista* (2010); *Azteca*/*Brachymyrmex* (2013); *Brachymyrmex*(2014); *Pheidole* (2015); *Crematogaster* (2017-2019) |
| 225520 | Alive | *Brachymyrmex* (2014-2015); *Crematogaster* (2017-2019) |
| 231681 | Alive | *Azteca* (2013); *Wasmannia* (2014-2015); *Myrmelachista* (2017-2019) |

**Fig. S2.** DBH and relative growth rates (RGR) comparisons over 12-years in a 50-ha plot in Yasuní National Park. In a (A) we show the DBH averages of host trees of *M. schumanni* and *Azteca* spp. throughout the 2007–2019 surveys. ±95% confidence intervals are shown. Linear regression equations and fitness tests of significance are given: *M. schumanni* (*y* = 2.885*x* + 49.569, R^2^ = 0.96, F = 99.15, *p* = 0.009); *Azteca* spp. (*y* = 1.251*x* + 54.072, R^2^ = 0.98, F = 40.51, *p* = 0.023). **(B)** RGR of host trees of *M. schumanni* and *Azteca* spp. throughout each time period. ±95% confidence intervals are shown.


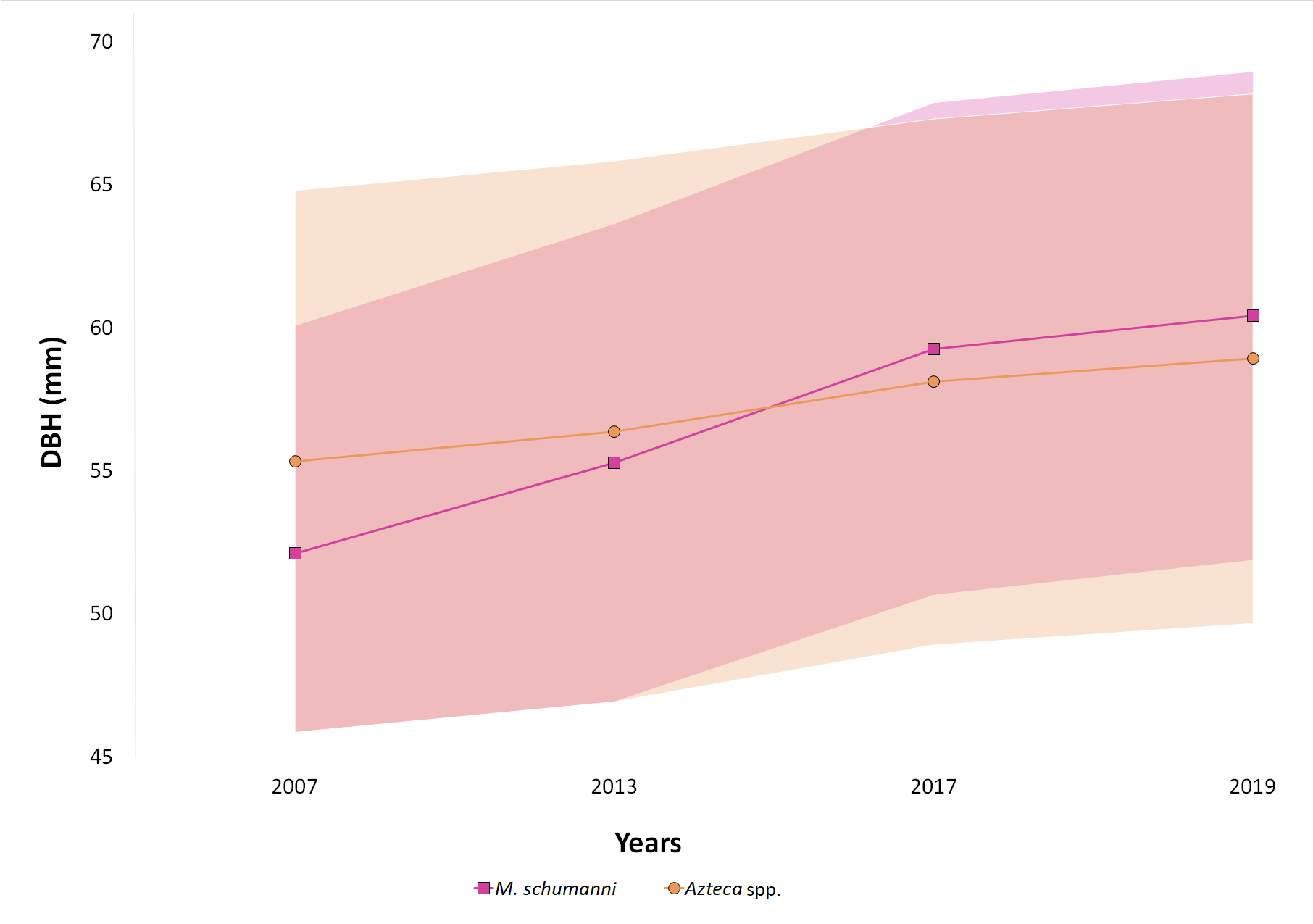

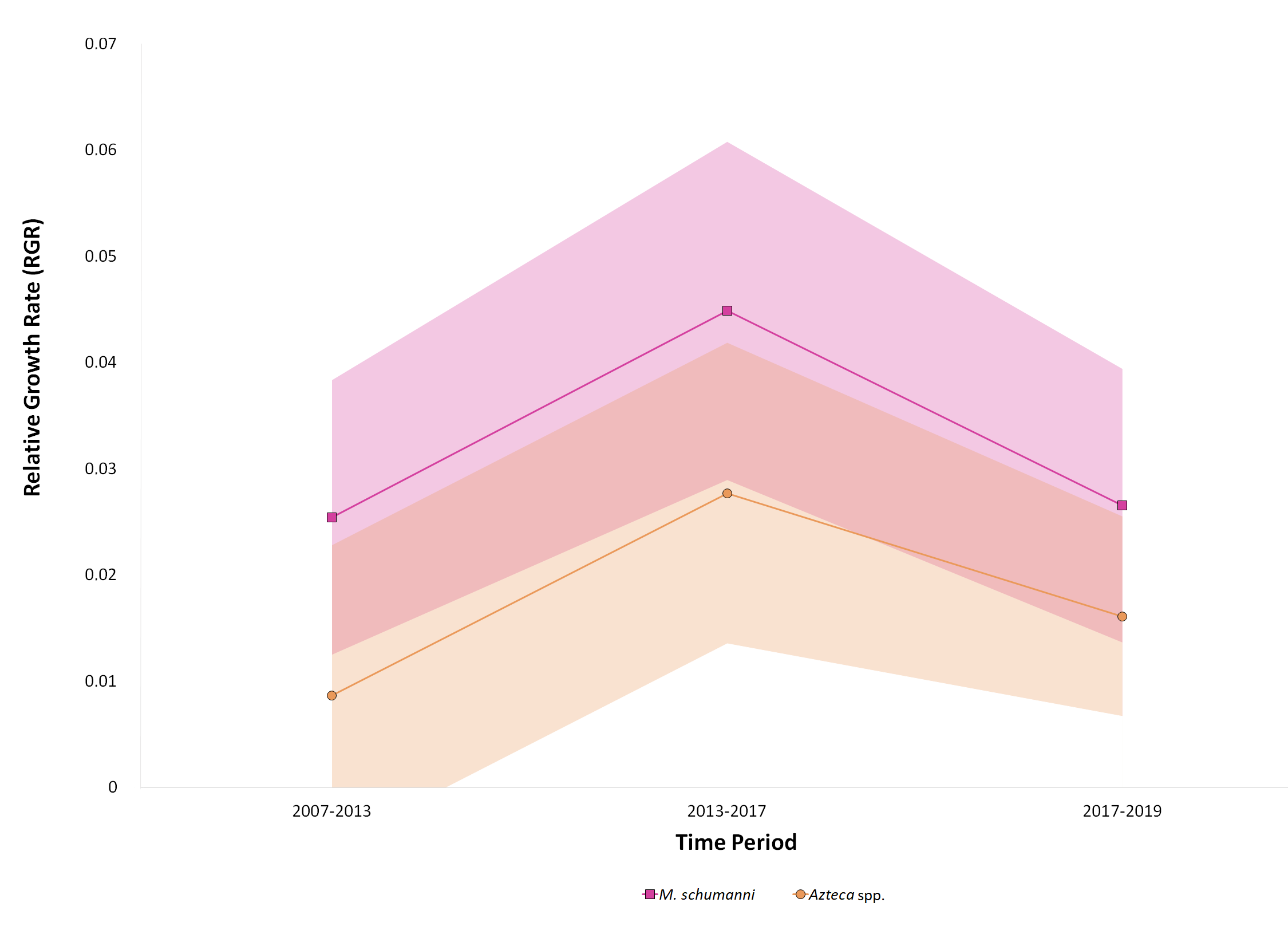

Supplement: Supplementary file 1 — Supplementary Information. [file 41598_2024_67140_MOESM1_ESM.docx]
